# Eigenvalue productivity: Measurement of individual contributions in teams

**DOI:** 10.1371/journal.pone.0273623

**Published:** 2022-09-15

**Authors:** Julia Müller, Thorsten Upmann

**Affiliations:** 1 School of Business Administration and Economics, University of Osnabrück, Osnabrück, Germany; 2 Helmholtz-Institute for Functional Marine Biodiversity at the University of Oldenburg (HIFMB), Oldenburg, Germany; 3 Institute for Chemistry and Biology of the Marine Environment, University of Oldenburg, Campus Wechloy, Oldenburg, Germany; 4 Faculty of Business Administration and Economics, Bielefeld University, Bielefeld, Germany; Universidad de Murcia, SPAIN

## Abstract

While the output of a team is evident, the productivity of each team member is typically not readily identifiable. In this paper we consider the problem of measuring the productivity of team members. We propose a new concept of coworker productivity, which we refer to as eigenvalue productivity (EVP). We demonstrate the existence and uniqueness of our concept and show that it possesses several desirable properties. Also, we suggest a procedure for specifying the required productivity matrix of a team, and illustrate the operational practicability of EVP by means of three examples representing different types of available data.

## 1 Introduction

Teams jointly produce a product. By judging the quality of this product, the success of the team as a whole can be easily assessed. However, the individual productivity of a worker is typically not directly observable, and thus it is difficult to conceptualize, even if a fixed task is assigned to each member of a team (see, *e.g.*, [1] and the references therein). This difficulty in assessing individual productivity is particularly significant if the concept is to be operationalizable with observable data. We, therefore, propose a new concept of coworker productivity to measure the individual contribution of a team member. We then demonstrate the existence and uniqueness of that concept and show that it has several desirable properties, may be applied in various situations, and is suitable for various types of data.

As is well-known, a team is more than merely the sum of its members’ individual abilities. Within a team, coworkers interact and their abilities as well as their capacities for and their productivities in teamwork determine, along with other factors, the actual team output—and thus the success of the team. In this way, the interplay of a worker with their teammates depends on the ability and actual willingness of the other coworkers to cooperate: a worker can only interact with the teammates efficaciously if they go along with this endeavor. (If cooperation and coordination is not successful, problems like free-riding can arise in teams, analyzed for example by [2–4]). As a consequence, the effective contribution of a worker to the team depends on the contributions of the others and thus on the cooperative capacity, the social competencies, the skills, and the efforts of the other coworkers. (The relevance of the amount of effort workers choose, based on income maximizing considerations, to put forth when on a team has been emphasized by [5]).

While this should be beyond controversy, it does not readily provide an operational concept of an individual’s contribution to the productivity of the team. Similarly, the coworkers’ productivities cannot be directly observed and are thus not readily derivable from the team data. While the productivity or success of a team as a whole is usually observable or relatively easy to measure, *e.g.*, by total sales, revenue, patterns, number of orders or cases, percentage of wins, points scored, etc., it is more difficult to measure the productivity of an individual worker within a team (see also [6]). This difficulty is inherently associated with the very nature of teamwork: while the team as a whole produces a joint output, the individual contributions of the team members can only be measured in terms of input, such as working time, effort, etc. Apparently, this is a consequence of the fact that “the interaction between team members is multifaceted” ([7, p. 4]), and this multiplicity of interactions raises the question of how one can consistently define and then calculate coworker productivities.

For example, in team sports, the literature has used numerous variables to appraise the coworker productivity of a player: the number of goals scored, assists provided, duels won, ball touches, etc. Yet, each of these numbers suffers from the fact that it imputes an output (successful action) to an individual player, while this output is actually the joint product of the player and the teammates: a player can only perform well when the other teammates are able and willing to put that player into the scene and lay the proper groundwork for the player’s actions to bear fruit. At the same time, professional team sports require, besides individual aptitudes and skills, complex collective moves, which constitute both a prerequisite for and a consequence of individual performance (see also [8]). Through all of these channels, the team productivity of a player depends on the productivities of all the teammates, and in particular on the productivity of that player’s “neighbors” on the playing field—accordingly, there is empirical evidence that some combinations of positions or some pairs of players are more strongly complementary than others (see, for example, [9])—and similar arguments apply to any complex team production process in economics. Due to this reciprocity the productivities of all team members are determined endogenously and simultaneously [6].

For the case of NBA teams, Berri [10] proposed an econometric model specifically tailored to basketball along with a related concept to measure the productivity of individual players. Furthermore, he criticized the use of seemingly straightforward shortcuts to measure individual team productivity, such as the number of points scored, as they are “likely to be incorrect and misleading” (p. 415) since they neglect the specific team sport aspect: the necessary cooperation between team members. We act on the idea put forth by [10] and develop a theoretical concept of coworker productivity which is quite general and flexible enough to be used in many different economic contexts and with many different available databases—and is thus applicable in various situations. In particular, it does not require a specific model or economic situation, as does Berri’s basketball-specific approach: all it requires is some (locally) linear structure and the pairwise decomposability of total productivity. This structure suffices to obtain a measure of coworker productivity enjoying convenient properties. In this way, our work follows a suggestion of [11], who called for more research “to derive measures of players’ ‘true’ marginal products when productivity spillovers exist” (p. 401).

In order to capture the prevalence of spillover effects and the interdependencies between the team productivities of the coworkers, a more sophisticated concept for measuring the contribution (or the relevance) of a coworker to the productivity of the team seems to be necessary. In this paper we, therefore, delineate a concept of coworker productivity which makes use of the reciprocal structure of cooperation within a team: in teams, coworkers are required to cooperate in order for the team to perform successfully, while each coworker also benefits from the cooperative abilities and social skills of the other teammates. Specifically, the more a teammate of coworker *i* contributes to the team, the better the conditions will be for coworker *i* to perform well—and thus to contribute to the success of the team. In this way, the team productivity of coworker *i* depends (positively) on the productivities of all other teammates and, in particular, on the productivity of those who are “adjacent to” or “central for” that player. Since this is true for any team member, coworker productivity is simultaneously reciprocal to all team members; and consequently, the productivities of all coworkers on a team must be determined simultaneously.

We duly acknowledge the feature of mutual complementarity in production within teams and present a concept of coworker productivity that consistently and simultaneously defines the contributions of each coworker to the productivities of all other teammates.—Formally, complementarity in production is captured by the supermodularity of a production function. For more details, see, for example, [12] or the excellent monograph by [13]. This supermodularity may vanish in the limiting case if members work on their own, as, for example, in the case of team competition with pairwise matches, a case analyzed by [14].—In this way, the concept serves to measure the cooperative complementarity of production, and thus the significance or centrality, of a coworker within a team. Formally, our concept of coworker productivity is defined as a vector that, when we apply a linear function to it (represented by a nonnegative matrix of pairwise productivity coefficients), does not have its direction changed. By construction, this concept is closely related to the concept of eigenvector centrality, introduced by [15]—in fact, the idea can even be traced back to [16], who proposed a similar index to measure the status of individuals within a group—which has been frequently applied in network analysis, notably by Google’s PageRank algorithm to rank search results in the Web [17]. In order to acknowledge that origin, we refer to our concept as *eigenvalue productivity* (EVP). Moreover, since EVP provides a ranking of the team members, our concept is also related to the literature on rankings. (For a short survey on spectral ranking, we refer the reader to [18]).

In our approach, we calculate the coworker productivities for a given team assigned to a given task. We thus do not model the decision (process) of the manager on the optimal composition of the team, or, more broadly, how or why a manager selected a specific worker for a given project. This decision is not an issue here, but is considered to be exogenously determined. Yet, in a more general framework, pairwise productivities may be chosen directly by the players, thereby establishing a pairwise cooperation game or, in this sense, a network game. These possibly action-dependent pairwise productivities of the coworkers are considered as given data here, because the central purpose of EVP is to derive a scalar productivity measure for each player when the actions have already been determined. There is, though, a significant literature that explores the activities chosen by the players for a given (possibly uncertain) network structure; we briefly discuss this in the literature review in Section 2.

While the concept of EVP is related to network analysis, the spirit of EVP also reflects the basic idea incorporated in the Shapley value, well known from cooperative game theory [19]: the distribution of the surplus between players according to each player’s marginal contribution to all possible coalitions. (This relationship to cooperative game theory has recently also been used by [20] when they consider the problem of sharing the revenues from broadcasting league tournaments.) However, since the Shapley value requires that one already knows a player’s marginal contribution to all possible coalitions, it is not helpful or applicable when measuring individual productivity within a team: while we can observe both the output of the team (with a fixed composition) and individual inputs, we cannot deduce the marginal contributions unless we know the production function for each possible coalition.

To measure the cooperative productivity of each team member—that is, the productivity effects a member generates in team production beyond their stand-alone productivity—the EVP concept relies on the pairwise interactions of all team members. These interactions, as multifaceted as they are, include both intrinsic and behavioral characteristics: willingness to cooperate, effort, commitment, communication, mutual understanding may all entail, to a lesser or greater extent, intrinsic as well as behavioral components. Without any additional information, we cannot disentangle these components: Quantifying intrinsic productivity is impossible in the absence of information on individual contributions, and vice versa (see, for example, [1, 6]). But once we have some additional information about either intrinsic or behavioral components of the team members, EVP can be used to compute the other component.

The approach that is presumably closest in spirit to our EVP concept is *CoScore* introduced by [6]. Both concepts use data of team membership and team production to delineate a measure of individual productivity in teams, where the productivity measures of all team members “are determined simultaneously and endogenously as a solution of a fixed point problem” ([6, p. 2]); that is, in both cases the productivity measure of each team member depends on the measures of their teammates. Yet, while EVP and *CoScore* rely on the same idea, they differ in their prerequisites (*CoScore* requires that each individual has at least one solo project), their construction (EVP uses a linear, *CoScore* a non-linear structure) and their properties (which are at considerable variance); and as a consequence both concepts are suitable in different situations; for example, in team sports individuals do, by definition, not perform solo projects so that *CoScore* is not even well defined in this context.

After providing the formal definition of EVP, we prove the existence and uniqueness of this concept and demonstrate that it has several economically desirable properties: *symmetry, permutation covariance, null player property, aggregate balance, differentiability, relative monotonicity, absolute monotonicity*, and *duplication monotonicity*. (Recently, [20], exploring the problem of sharing revenues from broadcasting in round-robin sport tournaments, also follow this type of an axiomatic approach; among other properties these authors postulate an *equal treatment of equals* and a *null team* property, which correspond to our *symmetry* and *null player property*, respectively.) Then we show how the EVP–vector can be calculated. Its calculation is based on the availability of a pairwise productivity matrix. If such a matrix of the directional productivity effect between each pair of members of a team is readily available, the EVP–vector can be directly employed. Frequently, though, this detailed data will not be available, and the pairwise productivity matrix has to be approximated or estimated from available data in a first step. In those cases, the EVP–vector can only be as good as the data is that is used in the computation of the productivity matrix. So, depending on the availability of data, we have to think about how to best deduce the pairwise productivity coefficients from available data.

We here suggest a procedure to calculate this productivity matrix from the data of a team and illustrate the operational practicability of EVP by means of three examples, one artificial and two real world examples: The first example represents a hypothetical situation that is very clear and instructive, the second example uses real data from the movie industry, and the third example we uses publication records of a group of researchers. (The latter two a relegated to the Appendix.) In all three examples, we manage to use the available data to delineate the productivity matrix and to calculate the EVP–vector of the team. Since the data sets are still manageable, all calculations can even be verified by hand. This procedure makes use of the available data by carefully deducing pairwise productivities. Apparently, the procedure we deploy is not the unique conceivable way to calculate the productivity matrix, others procedures may be equally reasonable; and the more data is available the better is the basis for its calculation.

## 2 Related literature

There is ample research on various aspects of teamwork in both economics and management. In the following, we focus on those aspects of teams and teamwork that are especially relevant for the measurement of individual contributions within a team and thus for EVP.

Investigating the decision-making process, the literature in experimental economics concludes that groups generally arrive at more rational decisions than do individuals (see the reviews [21, 22]), suggesting that teamwork outperforms individual actions. For example, [23, 24] who explore the differences between the learning and adjustment process of teams and that of individuals, identify positive synergies between teammates and demonstrate the superiority of team play; [25] find that teams arrive at better investment decisions; [26–28] show that group membership has a strong effect on individual decisions; and [29] demonstrate that teams coordinate more successfully than individuals. More recently, in their laboratory experiments [1] show that there are individuals, which they call *team players*, who, when added to a team, consistently lead to an outperformance of the team over its predicted performance, *i.e.*, “beyond what their individual task-specific skills would suggest” (p. 2639); moreover, these team players have higher social skills and seem to motivate teammates to exert more individual effort.

Similarly, the significance of productivity spillovers between coworkers within teams has been demonstrated in various economic domains: examples are, among others, [9, 11, 30–32]. Also, several field studies demonstrate the effects of peers on the productivity of workers, see for example [33–36], who confirm the presence of significant complementarity between team members. ([37] provide a theoretical analysis of the influence of peers on other workers.) Moreover, [38] compare results from laboratory experiments with field studies and conclude that both yield similar estimates of peer effects. Thus, even though the productivity of coworkers may significantly differ across different teams, the relevance of coworker and teammate effects on individual productivity is apparently an omnipresent phenomenon; and, as emphasized by [8], a similar observation holds for the significance of team composition.

Within a team, the interactions between its members depend on behavioral aspects, group membership effects, employment practices, and other factors, all of which are well documented in the literature: [39–42] evidence the (positive) significance of innovative employment practices and diversity on team production. Investigating hierarchical differentiation in pay and participation in professional sports, [43] find those differentiations may facilitate intra-group coordination and cooperation.

There is also much interest in the relationship between salaries, namely the wage disparities within a team, and team performance, which has been explored by, for example, [44, 45]. Also, intergroup competition and the payment scheme influence the performance of a team and its individuals [46]. Generally, the distinction and resemblance of individual versus team financial incentives has been frequently explored, see for example [47–49].

Investigating team composition and the organizational structure of a team, [50] conclude that certain team roles are more important for team performance than others. The recognition of a single member of a team will, via social influence processes, produce positive spillover effects on the performance of the other team members, as well as on overall team performance, and this is particularly the case when the distinguished individual has a central position in the team (see [51]). More broadly, teams with more centralized structures are generally more successful: centrality is beneficial for individual and team performance (see [52–54]).

The degree and type of centralization of an organizational unit, such as a team, is frequently analyzed by means of tools well known from network analysis. For example, the centrality of a team member may be measured by degree centrality, closeness centrality, betweenness centrality, or eigenvector centrality (see [55]). Subsequently, versions of eigenvector centrality have been adopted in, among others, regional economics to characterize spatial structures (*e.g.*, [56, 57]), financial economics to measure risk exposure resulting from interconnectedness (*e.g.*, [58, 59]), the social sciences to measure social segregation (*e.g.*, [60, 61]), and network analysis to measure the effects of coauthorship on the performance of scholars (*e.g.*, [62, 63]). However, the most well-known application of eigenvector centrality is the PageRank algorithm [17, 64].

Beyond this, the network structure may also be endogenized, where team members choose their links to and intensities of cooperation with the other team members. Notable examples for network games are [65–70], for theoretical; and [71–75] for experimental work on network games. Moreover, [76–81], among others, endogenize the network structure, presenting models of network formation where players decide upon the costly creation of pairwise links. For a comprehensive survey of the theoretical work and the literature on games involving economic and social networks, we refer the reader to [55, 82, 83] and the survey [84].

## 3 The approach

In this section, we develop a new concept of coworker productivity, EVP, and show how individual productivities may be consistently determined for all members of a team. We then prove the existence and uniqueness of a *vector of eigenvalue productivities* (EVP–vector), and demonstrate that this vector has convenient economic properties. As argued above, a worker benefits from the abilities of all teammates: the more a teammate of worker *i* contributes to the team, the better the conditions are for worker *i* to perform well and thus to contribute to the success of the team. In this way, the coworker productivity of worker *i* depends (positively) on the productivities of their teammates. We shall now formalize this idea and show how coworker productivities may be derived.

Consider a team consisting of a fixed set of *n* workers N={1,2,…,n}. We assume that the individual productivity of each worker i∈N is nonnegative. According to the argument provided above, we presume that the productivity depends linearly on the productivity of *i*’s teammates. The *coworker productivity of worker* *i* *in team* N, $p^{i}(N)$, is then defined as

$$\begin{array}{c} p^{i}\left(N\right)=\frac{1}{\lambda }\sum_{j\in N}g_{ij}\left(N\right)p^{j}\left(N\right)\;\forall i\in N, \end{array}$$ (1)

where $g_{ij}(N)\;≧\;0$ denotes the extent to which worker *i* benefits from the coworker productivity of worker *j*, and correspondingly $g_{ii}(N)\;≧\;0$ represents the idiosyncratic productivity of worker *i*, while λ > 0 is a strictly positive normalization factor. The parameter $g_{ij}(N)$ measures the productivity–enhancing effect that worker *j* exerts on worker *i*’s productivity, an effect that depends on *j*’s proficiencies, team skills, social competencies, willingness to cooperate etc., as well as on *i*’s comprehension and receptiveness for these contributions of *j*. Since these effects are generically not symmetric, we should not expect *g*_*ij*_ to be equal to *g*_*ji*_. By this mechanism, the coworker productivity of *i* depends on the coworker productivities of all the teammates, and since relation (1) holds for any worker i∈N, we arrive at the equation system (in matrix notation)

$$\begin{array}{c} p\left(N\right)\equiv \frac{1}{\lambda }\;G\left(N\right)\;p\left(N\right), \end{array}$$ (2)

where the vector of the coworker productivities is denoted by $p(N)\equiv \left(p^{1},\ldots ,p^{n}\right)(N)$, and the matrix of the coefficients measuring the extent to which the individual productivities affect each other by $G(N)\equiv {\left[g_{ij}(N)\right]}_{i,j\in N}\;≧\;0$. In order to avoid the meaningless case **G** = **0**, we assume that there is at least one worker whose productivity is positive when paired with another worker, so that *g*_*ij*_ > 0 for at least some pair (i,j)∈N×N, and thus **G** ≥ **0**. (We use the following notation: For any $x,y\in {\mathbb{R}}^{n}$ we write $x\;≧\;y\;:\Leftrightarrow \;x_{i}\;≧\;y_{i}\;\forall i=1,\ldots ,n$; $x\geq y\;:\Leftrightarrow \;x_{i}\;≧\;y_{i}\;\forall i=1,\ldots ,n$ and *x*_*i*_ > *y*_*i*_ for some *i* (that is **x** ≠ **y**); **x** > **y**: ⇔*x*_*i*_ > *y*_*i*_∀*i* = 1, …, *n*. Also, if ***x*** ≧ ***0***, we call **x** a *nonnegative* vector; if **x** ≥ **0**, a *nonnegative, non-zero* vector; and if **x** > **0**, a *positive* vector. The corresponding notation and wording is used for matrices. Finally, we write ${\mathbb{R}}_{+}^{n}\equiv \{x\in {\mathbb{R}}^{n}:x\;≧\;0\}$ for the nonnegative orthant of ${\mathbb{R}}^{n}$.)

For notational convenience, we subsequently suppress the team argument N, but it should be kept in mind that **G** and **p** depend on the team under consideration. Then, Eq (2) may be re-written as

$$\begin{array}{c} \lambda p=Gp\;\Leftrightarrow \;(G-\lambda I)p=0, \end{array}$$ (3)

where **I** denotes the identity matrix (of the proper rank, *i.e.*, of rank *n* in this case). For **p** ≠ **0**, the homogeneous system (3) has a solution (in **p**) if, and only if, det (**G**−λ**I**) = 0. But this is equivalent to λ being an eigenvalue of **G**, and **p** being the corresponding eigenvector. Since **p** is the vector of individual productivities that we want to determine, we refer to our concept of coworker productivities as *eigenvalue productivity*. Hence, assuming that all information relevant for the productivity measure is contained in the matrix **G**, we define:

**Definition 1** (Eigenvalue productivity (EVP)). Let the set of team members be N={1,…,n} with n∈ℕ. Let $G\equiv G(N)\equiv {\left[g_{ij}(N)\right]}_{i,j\in N}\geq 0$ denote a nonnegative, nonzero matrix—the matrix of pairwise, directional production coefficients—and and let *ρ*(**G**) denote the spectral radius of **G**. Let λ = *ρ*(**G**) be a maximal eigenvalue of **G**. We call an associated eigenvector **p**(λ) a *vector of eigenvalue productivities* (EVP–vector).

The concept of EVP thus uses the information on the productivity relations among the team members contained in **G**, and assigns each member a nonnegative real number, *viz.* assigns the team a nonnegative vector, which is unique upon multiplication by a constant (or upon scaling).

## 4 Existence and properties of eigenvalue productivity

In this section, we formalize our assumptions on **G**, demonstrate the existence and uniqueness of eigenvalue productivity, and show that EVP satisfies a set of welcome properties. Specifically, EVP nicely respects the following ideas: a player’s productivity is increasing in the productivity of their teammates; the productivity of a player who fails to contribute anything to a team’s success is zero; symmetric players are treated identically; and the productivity of a player decreases as there are other players who provide exactly the same productivity profile, and thus are perfect substitutes for the player.

### 4.1 Normalization of the productivity matrix

For convenience, we normalize *g*_*ii*_ = 1 for all i∈N, implying **G** ≥ **I**, We summarize this in the following assumption.

**Assumption 1**. *The matrix of pairwise productivity coefficients* **G** *is nonnegative, nonzero*, i.e., **G** ≥ **0**, *and irreducible with main diagonal elements being normalized to unity*, i.e., $g_{ii}=1,\forall i\in N$, *so that* **G** ≥ **I**.

Mathematically this normalization can be done w. l. o. g. (the next lemma provides a formal proof of this) so that we may set, more generally, $g_{ii}=c,\forall i\in N$, for some nonnegative constant *c*, and our assumption will be replaced by **G** ≥ *c* **I**; in particular, for *c* = 0, our assumption says that **G** is nonnegative and nonzero. In order to acknowledge the possibility of an arbitrary, nonnegative normalization of the diagonal elements, the definition of EVP (Definition 1) only requires **G** ≥ **0**, but for expository purposes, we will henceforth assume **G** ≥ **I**.

The main diagonal elements of the productivity matrix **G** represent stand-alone productivities (or fixed effects in econometric terms) of the team members; this also includes intrinsic (or idiosyncratic) components to the extent that they can be deployed on a stand-alone basis, *i.e.*, in solo projects. All team–dependent productivity effects, though, are captured by the off-diagonal elements of **G**. Since we want to conceptualize a measure of team productivity, we intentionally abstract from any heterogeneity among team members in team–independent productivities. For this reason, we deliberately disregard stand-alone productivities, and assume $g_{ii}=c,\forall i\in N$. We will discuss this issue in more detail in subsection 4.4.

**Lemma 1**. *Given some quadratic matrix* **A**, *let* **B** ≡ **A** + *α* **I** *with* α∈ℝ. *Then*, λ *is an eigenvalue of* **A** *and* **x** *is the associated eigenvector if, and only if*, *μ* = λ + *α* *is an eigenvalue of* **B** *with the associated eigenvector* **x**.

*Proof* By the definition of an eigenvalue and its associated eigenvector, we have **0** = (**A**−λ**I**)⋅**x** = (**A** + *α* **I**−(λ + *α*)**I**)⋅**x** = (**B**−*μ* **I**)⋅**x**

According to Lemma 1, a translation of the diagonal elements yields a corresponding translation of the eigenvalues but leaves the eigenvectors unchanged. For this reason, such a shift leaves the Perron vector unchanged (provided that **A** and **B** ≡ **A**−*α* **I** are both nonnegative). Consequently, we may choose any homogeneous normalization of the diagonal elements of **G**, *i.e.*, $g_{ii}=c,\forall i\in N$ for any *c* ≧ 0, without affecting the values of EVP. This is why we can w. l. o. g. set *c* = 1.

*Remark 1*. If *g*_*ij*_ = 0∀*i*, *j*, *i* ≠ *j*, we obtain from Eq (2) *g*_*ii*_ = (1/λ)*g*_*ii*_∀*i*, and thus λ = 1. But **G** = **I** (or, more generally, **G** = *c* **I** according to our previous remark) is an uninteresting case, of course, as in this case all (pairwise) team effects would be absent. Therefore, we assumed **G** ≥ **I** in Assumption 1.

### 4.2 Existence and uniqueness of the eigenvalue productivity

In this section, we use familiar results from linear algebra, *viz.* a version of the Perron–Frobenius theorem, to prove existence and uniqueness of EVP.

**Proposition 1** (Existence of EVP). *Let the set of team members be* N={1,…,n} *with* n∈ℕ. *For any nonnegative, nonzero* *n*×*n* *matrix of pairwise, directional production coefficients* **G** ≥ **0**, *there exists a nonnegative, nonzero vector of eigenvalue productivities* **p** ≥ **0**.

*Proof*. The proof follows from the existence of a nonnegative Perron vector for nonnegative matrices (see, *e.g.*, [85, p. 375, Theorem 4.B.2]). Then, since **p** ≠ **0** by definition of an eigenvector, we conclude that **p** ≥ **0**.

The next result establishes the uniqueness of EVP under the prerequisite that **G** is irreducible.

**Proposition 2** (Uniqueness of EVP). *For any nonnegative, irreducible* *n*×*n* *matrix of pairwise, directional production coefficients* **G**, *the EVP–vector* **p** ≥ **0** *is unique up to a scalar multiple*.

*Proof*. The proof follows from the uniqueness of the nonnegative Perron vector for irreducible matrices (see, *e.g.*, [85, p. 372, Theorem 4.B.1(iii)]).

By construction, EVP represents a surjective but non-injective linear mapping, so that the EVP–vector is uniquely defined (except for scaling); but reversely **G** cannot be inferred or recouped from EVP. If we had not assumed in Proposition 2 that **G** was irreducible, the Perron root $\hat{\lambda }$ would not necessarily have been a simple root, and the EVP–vector would not necessarily be unique. That is, **p** and **q** with **p** ≠ *θ* **q** for any θ∈ℝ can be eigenvectors associated with $\hat{\lambda }$.

By Assumption 1, it holds that **G** ≥ **I**, and thus **G** ≠ **I**, so the Perron root is larger than one.

**Corollary 1**. *Given the matrix* **G** ≥ **0** *and some normalization of the diagonal elements* *g*_*ii*_ = *c* ≧ 0, *the Perron root of* **G** *satisfies* $\hat{\lambda }>c$. *In particular, for the normalization* *g*_*ii*_ = 1, *we have* $\hat{\lambda }>1$.

*Proof*. The proof follows from the fact that *g*_*ii*_ = *c* ≧ 0 implies **G** ≥ *c* **I** and from Theorem 1.5 in conjunction with Corollary 1 in [86].

Finally, if **G** is an irreducible nonnegative matrix, then **G** is primitive if it has at least one nonzero diagonal element Since we normalized the diagonal elements to unity—or, more generally, to some positive constant *c* > 0—any irreducible productivity matrix **G** is also primitive. Hence, we may apply the Perron–Frobenius theorem for primitive matrices, (see, *e.g.*, [86, p. 3f] and [85, p. 378, Theorems 390 4.B.3–4.B.5]).

### 4.3 Properties of eigenvalue productivity

We use the following notation. We write **x**_(−*i*)_ ≡ (*x*_1_, …, *x*_*i*−1_, *x*_*i* + 1_, …*x*_*n*_), and similarly we write **A**_(−*i*)_ for the matrix generated from **A** by removing column *i* and row *i*. Also, let **x**_(−[*i*])_ ≡ (*x*_*i* + 1_, …*x*_*n*_) denote the vector after removal of the first *i* components. For any two vectors **x** and **y**, we write 〈**x**, **y**〉 to denote their (standard) inner product. We write **e**_*i*_ to denote the *i*th unit vector of length *n*; and **1** to denote the all–ones vector (1,…,1)^⊤^ of length *n*. Also, let *π* be a permutation of the team members (*i.e.*, a one-to-one function from N to itself), and let *π*(**G**) represent the correspondingly permuted matrix where the rows and the columns of **G** are permuted as specified by *π*. In particular, *π*_*ij*_ denotes the *minimal permutation* where only the indices of players *i* and *j* are permuted, *i.e.*, $\pi_{ij}:N\to N$ with *π*_*ij*_(*i*) = *j*, *π*_*ij*_(*j*) = *i* and *π*_*ij*_(*k*) = *k* for all *k* ≠ *i*, *j*. This allows us to define the *i*–*j* *permutation equality* of vectors **x** and **y** by $x{≗}_{ij}y:\Leftrightarrow \pi_{ij}(x)=y\Leftrightarrow \pi_{ij}(y)=x$.

Let **g**_*i*•_ denote the *i*th row of matrix **G**; and **g**_•*j*_, the *j*th column of **G**. We say that players *i* and *j* *contribute equally in total terms*, if 〈**g**_*i*•_, **1**〉 = 〈**g**_*j*•_, **1**〉. A player i∈N who is contributing nothing to the productivity of the other team members, *i.e.*, $g_{i•}^{⊤}=e_{i}$, is referred to as a *null player*. Let $M(i)\equiv \{j|g_{j•}{≗}_{ij}g_{i•},j\in N\}$ denote the set of players with (after suitable permutations) identical pairwise productivities as player *i*, including player *i*. We refer to a player *j* ∈ *C*(*i*)≡{*j*|*g*_*ij*_ = *g*_*ji*_ = 0, *j*∈ *M*(*i*)\{*i*}} as a *clone* of *i*, and to *C*(*i*) as the set of clones of *i*. Using this notation, we formalize the following properties for a productivity measure ***φ***, or axioms that we may want a productivity measure to satisfy, and then show that EVP has those properties.

**Definition 2**. A productivity measure $\varphi :{\mathbb{R}}_{+}^{n\times n}\to {\mathbb{R}}_{+}^{n}$ satisfies

**symmetry** if for two players i,j∈N, $g_{i•}{≗}_{ij}g_{j•}$, then ***φ***_*i*_ = ***φ***_*j*_.
**permutation covariance** for any permutation *π*: *π*(***φ***(**G**)) = ***φ***(*π*(**G**)).
**null player property** if player i∈N is a null player, then ***φ***_*i*_ = 0.
**aggregate balance** if all players contribute equally in total terms, then ***φ***_*i*_ = ***φ***_*j*_, ∀i,j∈N.
**differentiability** ***φ*** is differentiable with respect to the team coefficients *g*_*ij*_.

The properties *symmetry* and *permutation covariance* represent quite natural properties of a productivity measure. (Property *permutation covariance* can be equivalently expressed as follows. Suppose *π* is a permutation. Let **P** = [*p*_*ij*_] be the permutation matrix defined by *π*, *i.e.*, *p*_*π*(*j*)*j*_ = 1, *j* = 1, …, *n*, and *p*_*ij*_ = 0, for all *i* ≠ *π*(*j*). Then $P^{⊤}\varphi (G)=\varphi (P^{⊤}GP)$.) In particular, *symmetry* requires that players contributing equally to each team member should be treated identically by the productivity measure, while *permutation covariance* demands that a renumbering (or renaming) of the players should not affect their productivity measures. Thus, upon renumbering the players, the productivity measures change accordingly, and in this sense the productivity measure is covariant with that renumbering.

The *null player property* states the natural requirement that a player contributing nothing to a team should be assigned a productivity measure of zero. The property of *aggregate balance* postulates that if *all* players contribute equally in *total* terms, then the same productivity measure should be assigned to each team member irrespective of the distribution of their pairwise productivities. This property does not require, though, that two players contributing in equal total terms have the same productivity measure, unless all other *n*−2 players also contribute the same total amounts (or unless, due to *symmetry*, the two players contribute equally to each player). *Differentiability* is an economically reasonable and mathematically convenient property implying that small productivity changes do not bring about abrupt changes in the productivity measure: rather, small perturbations of the productivity matrix lead to small changes in the productivity measure. (The results for the derivatives of the Perron vector with respect to the matrix elements can be found, for example, in [87, 88].)

In addition, we define the following monotonicity properties.

**Definition 3**. A productivity measure $\varphi :{\mathbb{R}}_{+}^{n\times n}\to {\mathbb{R}}_{+}^{n}$ satisfies

**relative monotonicity** if the *i*th row of **G** is perturbed by $\text{d}g_{i•}\geq 0^{⊤}$, then the relative productivity measure of player *i*, ***φ***_*i*_/***φ***_*j*_, ∀*i*, *j*, *i* ≠ *j*, does not decrease.
**absolute monotonicity** normalize ***φ*** such that ***φ***_*i*_ = 1, then a nonnegative, nonzero perturbation of the *i*th row of **G** by $\text{d}g_{i•}\geq 0^{⊤}$ does not increase the productivity of any player j∈N, *j* ≠ *i*, *i.e.*, $\varphi (G)\;≧\;\varphi (G+e_{i}g_{i•})$.
**duplication monotonicity** if player j∈N is a clone of player i∈N, then $\varphi_{i}(N)≦\varphi_{i}(N\backslash \{j\})$.

The properties *relative monotonicity* and *absolute monotonicity* characterize the behavior of the productivity measure if the pairwise production coefficients of player *i* (weakly) increase; that is, they characterize the effects of nonnegative (nonzero) perturbations of the *i*th row of **G**. Naturally, since a nonnegative, nonzero perturbation of the *i*th row of **G** means that player *i* becomes more productive than at least one other player, we should expect the productivity measure to respect this increase in productivity. In particular, *relative monotonicity* and *absolute monotonicity* say that, after suitable normalization, the (relative) productivity measure of player *i* should not decrease in response to an increase in the pairwise contributions of that player.

Finally, *duplication monotonicity* states that if we add a clone of player *i* to the team N, the productivity measure of player *i*, and thus of all players of type *i*, *i.e.*, all players in *M*(*i*), does not increase. Loosely speaking, enlarging a team by adding clones (weakly) decreases the productivity measure of all players whose characteristics are duplicated. Intuitively, the more clones of player *i* the team contains, the less crucial or the more dispensable this type of a player becomes.

**Proposition 3** (Properties of EVP). *Let* **G** *satisfy Assumption 1. Then, EVP satisfies* symmetry, permutation covariance, null player property, aggregate balance, differentiability, relative monotonicity, absolute monotonicity, *and* duplication monotonicity.

*Proof*. The proof is relegated to S1 Appendix.

EVP thus has a set of desirable properties. Specifically, it is a remarkable observation that while the Perron root of **G** is a non-decreasing function of positive perturbations, the components of the corresponding (normalized) Perron vector are non-increasing. Since this translates into a relative increase in the EVP value of the player whose row-entries are weakly increased, this is a plausible, and even natural feature of EVP. In view of *permutation covariance*, the reciprocal or self-referential structure, and the linearity of EVP, it is quite intuitive that it also satisfies *aggregate balance*: if all players contribute by the same total amount to the team, the same productivity measure will be assigned to all players. Notably, the reverse also holds, so that we infer:

**Corollary 2**. *The EVP values of all players coincide if, and only if, all players have equal total productivities*.

$$\forall i,j\in N:\left\langle g_{i•},1\right\rangle =\left\langle g_{j•},1\right\rangle \;\Leftrightarrow \;p=\alpha \;1,\;\alpha >0.$$

*Proof*. Since **p** is an eigenvector and $\hat{\lambda }$ the associated eigenvalue of **G**, we have by Eq (2), $G\cdot \alpha \;1=\hat{\lambda }\;\alpha \;1\;\Leftrightarrow \;G\cdot 1=\hat{\lambda }\;1\;\Leftrightarrow \;\langle g_{i•},1\rangle =\hat{\lambda },\forall i=1,\ldots ,n$.

While a sufficient condition for two players having the same productivity measure is that, by *symmetry*, both players equally contribute to the productivities of all players, equal pairwise contributions are not necessary for two players to have the same EVP value.

**Example 1**. A simple example is the matrix

$$G=(\begin{array}{cccc} 1 & 0 & 2 & 2\\ 0 & 1 & 1 & 1\\ 0 & 2 & 1 & 0\\ 1 & 0 & 0 & 1 \end{array})$$

with eigenvalues {3, −1, 1, 1}, and therefore $\hat{\lambda }=3$, and an associated eigenvector $p(\hat{\lambda })={\left(2,1,1,1\right)}^{⊤}$. Hence, players 2, 3, and 4 all have the same EVP value although their pairwise contributions do not coincide. Also, this example illustrates that the reverse implication of *aggregate balance* does not hold: equal total contributions are not necessary for two players to have equal productivity measures; players 3 and 4 have the same EVP value although their total contributions do not coincide: $G\cdot 1={\left(5,3,3,2\right)}^{⊤}$.

The *null player property* not only ensures that such a player obtains an EVP value equal to zero, but also that we may add or remove null players from the team without affecting the EVP values of the other players (including other null players).

**Corollary 3**. *A null player can be removed from (or added to) a team without affecting the EVP value of any player, that is*, **p**_(−*i*)_(**G**) = **p**(**G**_(−*i*)_).

*Proof*. This result follows from the proof of Proposition 3, see Eq (5) in S1 Appendix.

Comparing the EVP values of the teams N and N\{i} where *i* is a dummy player, we see from Corollary 3 that **p**(**G**) and **p**(**G**_(−*i*)_) only differ by the element *p*_*i*_ = 0 (the EVP value of the dummy player), which is contained in **p**(**G**) but not in **p**(**G**_(−*i*)_). In this sense, the EVP–vector of the team is unaffected by the removal (or the addition) of null players.

### 4.4 Acknowledging stand-alone productivities

Any worker’s contribution to a joint product consists of two parts: the stand-alone productivity and the team productivity of the worker. The former represents the productivity in solo projects, while the latter captures all interactions in production between that worker and the other team members—and it is this latter productivity which we are interested in here. We may regard the stand-alone productivity, which arguably consists of proficiency, knowledge, experience, efficiency, discipline, tenacity etc. as the *intrinsic* (or idiosyncratic) productivity of a worker, and the social competencies, such as the willingness to cooperate, communication skills, flexibility, obligingness etc., as constituents of team productivity. However, the latter characteristics are to some extent also intrinsic, it is just that these characteristics can only be observed in the context of a team but are unobservable in a solo project. Due to this conceptual difficulty, we prefer to speak of stand-alone productivity and team productivity.

Since, in this paper, we are interested in conceptualizing and measuring team productivities, we intentionally disregard any heterogeneity in stand–alone productivities, which is reflected in our assumption of homogeneous diagonal elements of **G**, *i.e.*, $g_{ii}=c,\forall i\in N$. Yet, by relaxing this assumption and specifying a non-constant main diagonal, we may include stand-alone productivities. In order to be able to calculate the stand-alone productivities, the required team data has to be structurally richer, because it has to include data for singleton teams; that is, projects that are performed by a single person (solo-projects). By assuming a homogeneous main diagonal of the productivity matrix, though, this data is not a prerequisite for EVP, unlike *CoScore* of [6].

Provided that data for the stand-alone productivities is available, we may genaralize EVP and substitute the constant diagonal (*c*, …, *c*) by generically non-homogeneous stand-alone productivities (*g*_11_, …, *g*_*nn*_). This substitution brings about the expected monotonicity effects: Any increase of stand-alone productivity of player *i*, *i.e.*, an increase of *g*_*ii*_, favors, *ceteris paribus*, the EVP of player *i* both in absolute and in relative terms due to *relative monotonicity* and *absolute monotonicity*.

## 5 Calculation of eigenvalue productivity

In the previous section we defined EVP and discussed its the properties. It is straightforward to calculate coworker productivities, as measured by EVP, for the members of a given team, provided that the matrix **G** is available. In real world applications, though, we frequently cannot directly observe the (marginal) effect of worker *i* on worker *j*’s productivity, so that the productivity matrix **G** is not readily available, but has to be calculated from available data. In this section, we demonstrate how this can be done, and then we employ three examples to illustrate the applicability of our approach. The first example, which is presented in sub-section 5.2, is a small artificial, yet very instructive example; it demonstrates how the matrix **G** and the EVP–vector can be calculated, and that the latter concurs with differences in productivities of the team members that are apparent from a close look at the data. Two other examples use real-world data to calculate the EVP–vectors for somewhat larger teams, thereby demonstrating the applicability of EVP on different domains. Specifically, the second example, which can be found in S2 Appendix, is a real-world example where we consider a group of directors, actors and directors of photography who work together to produce movies; the third example, provided in S3 Appendix, consists of a group of researchers who collaborate on different co-authorships in journal publications. These two real-world examples show that EVP works quite well on available data.

In all three cases, we have a situation where teams work in different compositions to realize a project, *e.g.*, movies or publications, and where a suitable measure of success is available. Variations in the team composition and in the success of the project are essential, as those allow us to calculate the matrix **G**. In fact, a sufficient, yet not excessive, variation in the team composition and nonnegative pairwise productivity coefficients are the only requirements on the data to make EVP work.

### 5.1 Calculation of the matrix G

First, delete from the team, *i.e.*, from the set N, those workers who have never been in action during the given period. Then, calculate the entries of **G** as follows: For each given unordered pair of workers {i,j},i,j∈N, consider those projects in which *i* and *j* have worked together, that is, where both were included in the team composition. Calculate the ratio of the points (which we use as a generic term for any success measure) achieved in these projects to the maximal number of points the teams including the pair {*i*, *j*} could have potentially achieved. Let *s*_*ij*_ denote this point ratio, which measures the success (performance) of the pair {*i*, *j*} over all team compositions. Next, consider those projects where worker *i* was a member of the team (worker *j* may or may not have been a member of the team), and calculate the points the respective teams have achieved in these projects divided by the maximal number of points those teams could have potentially achieved; denote this point ratio by *s*_*i*_ ≡ *s*_*ii*_, which measures the success (performance) of worker *i*. As a convention, for pairs of workers {*i*, *j*} that have never been jointly included in some team composition during the period, we set $s_{ij}=\sqrt{s_{i}s_{j}}$. Collecting all pair-success ratios gives the symmetric matrix $S\equiv {\left(s_{ij}\right)}_{i,j\in N}$.

Next, we define the ratio *g*_*ij*_ ≡ *s*_*ij*_/*s*_*j*_, i,j∈N, which represents the relative performance of the pair {*i*, *j*} compared to the overall performance of worker *j*, an effect which can be attributed to the cooperation with worker *i*. With this definition, the main diagonal elements equal unity, *i.e.*, $g_{ii}=1,\;\forall i\in N$. The elements of the *i*th row of **G** capture the increase in the productivity of each worker *j* due to the contribution of worker *i*. Inversely, the *i*th column of **G** represents how each of the team members contributes to the performance of worker *i*. In this way, the quadratic, non symmetric matrix **G** represents all relative normalized pairwise performance measures.

### 5.2 Example 1: A simple example

Consider a team consisting of five workers *N* = {*A*, *B*, *C*, *D*, *E*}. Assume that over a specific time period the team has put into effect 17 projects in six different compositions, and has realized 26 out of 51 possible (abstract) points of success. The detailed results for the specific team compositions are displayed in Table 1.

**Table 1:** Results of the team for varying compositions.

no.	compositions	projects	points	max. pts.	ratio
1	*ACD*	2	3	6	$\frac{1}{2}$
2	*ACE*	4	12	12	1
3	*ADE*	3	1	9	$\frac{1}{9}$
4	*BCD*	3	4	9	$\frac{4}{9}$
5	*BCE*	2	6	6	1
6	*BDE*	3	0	9	0
	sum	17	26	51	$\frac{26}{51}$

Before we proceed to calculate the EVP value of all workers, it is worthwhile to pause for a second and to inspect Table 1 for the individual contributions of the workers. Apparently, the success of the team has improved whenever *C* joined the team: compare the composition *ADE* with *ACD*, *ADE* with *ACE*, *BDE* with *BCD*, and *BDE* with *BCE*. In all of these comparisons, the ratio of achieved points to maximal points has gone up by replacing either *D* or *E* by *C*. It thus appears that the coworker productivity of *C* is relatively high—and this should be acknowledged by the EVP of *C*. Workers *A* and *B* were never included in the same team composition, a situation which may occur for workers with the same area of expertise, *e.g.*, IT specialists or goalies in team sports. It is easy to verify that the team performance has improved whenever *B* has been replaced by *A*; and that the team performance has declined whenever *D* has been included. Accordingly, the EVP should assign a higher coworker productivity to *A* than to *B*, and it should assign worker *D* a particularly poor coworker productivity.—We shall see, EVP exactly reflects this.

From Table 1 we calculate the results for each worker *i* by disregarding those projects where *i* was not included in the composition. The individual results are shown in Table 2. In the next step, we have to calculate the success for each pair of workers {*i*, *j*}, *i*, *j* ∈ *N*, which is done in Table 3. Using that data we build the matrix **S**, the matrix of pairwise success, and then calculate **G** as described in the last subsection:

$$\begin{array}{c} S=(\begin{array}{ccccc} \frac{16}{27} & \frac{2\sqrt{5}}{9} & \frac{5}{6} & \frac{4}{15} & \frac{13}{21}\\ \frac{2\sqrt{5}}{9} & \frac{5}{12} & \frac{2}{3} & \frac{2}{9} & \frac{2}{5}\\ \frac{5}{6} & \frac{2}{3} & \frac{25}{33} & \frac{7}{15} & 1\\ \frac{4}{15} & \frac{2}{9} & \frac{7}{15} & \frac{8}{33} & \frac{1}{18}\\ \frac{13}{21} & \frac{2}{5} & 1 & \frac{1}{18} & \frac{19}{36} \end{array}),\;G=(\begin{array}{ccccc} 1 & \frac{8}{3\sqrt{5}} & \frac{11}{10} & \frac{11}{10} & \frac{156}{133}\\ \frac{3\sqrt{5}}{8} & 1 & \frac{22}{25} & \frac{11}{12} & \frac{72}{95}\\ \frac{45}{32} & \frac{8}{5} & 1 & \frac{77}{40} & \frac{36}{19}\\ \frac{9}{20} & \frac{8}{15} & \frac{77}{125} & 1 & \frac{2}{19}\\ \frac{117}{112} & \frac{24}{25} & \frac{33}{25} & \frac{11}{48} & 1 \end{array}). \end{array}$$ (4)

**Table 2:** Individual results for each worker.

worker	incl. in composition	points	max. pts.	*s*_*i*_
*A*	{1, 2, 3}	16	27	$\frac{16}{27}$
*B*	{4, 5, 6}	10	24	$\frac{5}{12}$
*C*	{1, 2, 4, 5}	25	33	$\frac{25}{33}$
*D*	{1, 3, 4, 6}	8	33	$\frac{8}{33}$
*E*	{2, 3, 5, 6}	19	36	$\frac{19}{36}$

**Table 3:** Pair results.

pair	incl. in composition	points	max. pts.	*s*_*ij*_
{*A*, *C*}	{1, 2}	15	18	$\frac{5}{6}$
{*A*, *D*}	{1, 3}	4	15	$\frac{4}{15}$
{*A*, *E*}	{2, 3}	13	21	$\frac{13}{21}$
{*B*, *C*}	{4, 5}	10	15	$\frac{2}{3}$
{*B*, *D*}	{4, 6}	4	18	$\frac{2}{9}$
{*B*, *E*}	{5, 6}	6	15	$\frac{2}{5}$
{*C*, *D*}	{1, 4}	7	15	$\frac{7}{15}$
{*C*, *E*}	{2, 5}	18	18	1
{*D*, *E*}	{3, 6}	1	18	$\frac{1}{18}$

Finally, we have to calculate the eigenvalues of **G**: its (absolutely) largest eigenvalue is $\hat{\lambda }=4.97$, and the associated eigenvector is (after normalizing by the first component) $p(\hat{\lambda })=\left(1,0.7849,1.3276,0.4492,0.9203\right)$—which is the EVP–vector we have been looking for. As appraised earlier when inspecting Table 1, the EVP values of worker *A* and *C* are high, while those of *B* and *D* are low. So, EVP reflects our intuitive notion of coworker productivity.

## 6 Discussion and conclusion

A classic problem in labor and personnel economics is: how can one measure individual contributions to a team or a group? Owing to the fact that a team’s output is, by definition, produced jointly, and frequently simultaneously, coworker productivity, *i.e.*, individual contributions to the joint output, is both difficult to conceptualize and hard to measure. In this paper, we contribute to resolving both issues. We first derived a new concept to measure coworker productivity, which we referred to as “eigenvalue productivity” (EVP), as it is built upon eigenvector centrality, a concept well established in network analysis. Then, we showed that under rather mild regularity conditions, such an EVP–vector, measuring the contributions of all team members, exists and is unique; and we demonstrated that this vector has convenient economic properties, namely *symmetry, permutation covariance, null player property, aggregate balance, differentiability, relative monotonicity, absolute monotonicity*, and *duplication monotonicity*. Finally, using data of team success—one from an artificial and two from real-world examples—we demonstrated how the productivity matrix can be generated from the compositions of a team and its performance over a fixed time period, such that EVP can then be calculated.

Although, the concept of EVP is quite general and flexible, there are some limitations on its applicability: First, EVP presumes that the pairwise productivities are nonnegative, *i.e.*, that there is a weak complementarity between all team members. In view of the strong empirical evidence in favor of complementarity within teams, though, this presumed complementarity should not constitute any material limitation of the concept. In fact, if some pairwise productivities were negative, implying the presence of some type of impediments between team members, one should consider disbanding the team. Second, if the matrix of pairwise productivities is not readily available, it must be calculated from available data. In this case, the EVP–vector obtained can only be as good as the data used in the computation of the productivity matrix is. Frequently, available data does not contain any information on pairwise effects, so that a suitable estimator for pairwise productivities has to be found. For this purpose, the data must comprise at least some, but not excessive, variety in team composition; as with data from either fixed team compositions or from completely disjunct teams, pairwise productivities cannot be inferred; this seems to be a rather weak requirement on the data, though.

In sum, we conclude that EVP is (i) a properly defined concept, which has several desirable properties; (ii) is suitable for most real-world situations of team composition in labor economics, personnel economics and team sports economics; and (iii) is applicable to a large variety of empirical data. We are therefore confident that EVP not only constitutes a significant theoretical contribution, but may also help calculate coworker productivities in applied empirical work.

## Supporting information

S1 Appendix Proof of Proposition 3 [86, 89, 90]. (PDF)

S2 Appendix Example 2: Success of teams in movies [91, 92]. (PDF)

S3 Appendix Example 3: A research group [93–97]. (PDF)
