# Supplementary material for: Eigenvalue productivity: Measurement of individual contributions in teams
Source: PLoS One. 2022 Sep 15;17(9):e0273623. doi: 10.1371/journal.pone.0273623 (PMC9477377; doi:10.1371/journal.pone.0273623)
Supplement: S1 Appendix — (PDF) [file pone.0273623.s001.pdf]

S1 Appendix. Proof of Proposition 3.

Proof. Proof of *symmetry*. Let two players have identical pairwise productivities.

W. l. o. g. we permute the ordering of these players so that we have for players 1 and 2: $\mathbf{g}_{1\bullet} \overset{\circ}{=}_{12} \mathbf{g}_{2\bullet}$. Assume, on the contrary, $p_1 = p_2$. We obtain from the first two rows of Eq. (3) $\hat{\lambda} p_1 = \langle \mathbf{g}_{1\bullet}, \mathbf{p} \rangle$ and $\hat{\lambda} p_2 = \langle \mathbf{g}_{2\bullet}, \mathbf{p} \rangle$. Subtracting both equations and using $g_{12} = g_{21}$ yields

$$(1 - g_{12})\, p_1 + (g_{12} - 1)\, p_2 = \hat{\lambda}\,(p_1 - p_2) \quad \Leftrightarrow \quad \left(\hat{\lambda} - 1 + g_{12}\right)(p_1 - p_2) = 0.$$

Since by Lemma 1 $\hat{\lambda} > 1$, and $g_{12} \geqq 0$, this is impossible, and thus $p_1 \neq p_2$ implies a contradiction.

Proof of *permutation covariance*. We know that a matrix $\mathbf{A}$ and its transpose $\mathbf{A}^\top$ have the same eigenvalues. For any given eigenvalue of $\mathbf{A}$ (and thus of $\mathbf{A}^\top$) λ_i, let $\mathbf{x}_i$ and $\mathbf{y}_i$ represent the corresponding eigenvectors of $\mathbf{A}$ and $\mathbf{A}^\top$, respectively; also let $\mathbf{X}$ denote the matrix that has $\mathbf{x}_i$ as its ith column, and let $\mathbf{Y}^\top$ denote the matrix that has $\mathbf{y}_i$ as its ith row. Then, since $\mathbf{X}^{-1} = \mathbf{Y}^\top$, we have $\mathbf{X}^{-1}\mathbf{A}\mathbf{X} = \mathbf{Y}^\top \mathbf{A}\mathbf{X} = \operatorname{diag}(\lambda_i)$. Since $\mathbf{T}^{-1}\mathbf{A}\mathbf{T}$ and $\mathbf{T}\mathbf{A}\mathbf{T}^{-1}$ both represent similarity transforms of $\mathbf{A}$, it is straightforward to show that the eigenvalues are invariant under a similarity transformation and that the eigenvectors are multiplied by $\mathbf{T}^{-1}$ (see [89, Ch. 1.5, p. 6f]). Then, if $\mathbf{T}$ is restricted to a permutation matrix $\mathbf{P}$, it follows that the eigenvectors are also permuted according to the permutation represented by $\mathbf{P}$.

Proof of *null player property*. W. l. o. g. we permute the ordering of the players such that the null player gets index 1. Then,

$$\mathbf{G} = \begin{pmatrix} 1 & \mathbf{0}^\top \\ \mathbf{b} & \mathbf{G}_{(-1)} \end{pmatrix}.$$

If $\mathbf{p}$ is the eigenvector associated with $\hat{\lambda}$, we obtain from Eq. (3)

$$\begin{aligned} \mathbf{0} &= \left(\mathbf{G} - \hat{\lambda}\mathbf{I}_n\right) \cdot \mathbf{p} \\ &= \left(\begin{pmatrix} 1 & \mathbf{0}^\top \\ \mathbf{b} & \mathbf{G}_{(-1)} \end{pmatrix} - \hat{\lambda} \begin{pmatrix} 1 & \mathbf{0}^\top \\ \mathbf{0} & \mathbf{I}_{n-1} \end{pmatrix} \right) \cdot \begin{pmatrix} p_1 \\ \mathbf{p}_{(-1)} \end{pmatrix} \\ &= \begin{pmatrix} 1 - \hat{\lambda} & \mathbf{0}^\top \\ \mathbf{b} & \mathbf{G}_{(-1)} - \hat{\lambda}\mathbf{I}_{n-1} \end{pmatrix} \cdot \begin{pmatrix} p_1 \\ \mathbf{p}_{(-1)} \end{pmatrix} \\ \Longleftrightarrow \quad p_1 &= 0 \wedge \left(\mathbf{G}_{(-1)} - \hat{\lambda}\mathbf{I}_{n-1}\right) \cdot \mathbf{p}_{(-1)} = \mathbf{0}, \end{aligned} \tag{5}$$

where we again used the fact that $\hat{\lambda} > 1$ by Lemma 1.

Proof of *aggregate balance*. If $\mathbf{G}$ has a constant row sum of μ, we have $\mathbf{G} \cdot \mathbf{1} = \mu \mathbf{1}$, implying that μ is an eigenvalue of $\mathbf{G}$ and $\mathbf{1}$ is the associated eigenvector. Since $\mathbf{G}$ is irreducible, μ equals the Perron root $\hat{\lambda}$ (alternatively, this follows from Theorem 1.5 in conjunction with Corollary 1 of [86]) and its associated eigenvector is unique (up to a scalar multiple).

Proof of *differentiability*. A proof of the differentiability of a simple eigenvector with respect to the entries of the matrix can be found in [89, pp. 66-77].

Proof of *relative monotonicity*. See Theorem 2.1 and the proof thereof in [90].

Proof of *absolute monotonicity*. See Theorem 3.1 and the proof thereof in [90].

Proof of *duplication monotonicity*. By definition of a clone, we have $\mathbf{g}_{1\bullet} \mathrel{\mathring{=}}_{ij} \mathbf{g}_{2\bullet}$ with the first two elements being 1 and 0 (and the same holds for the first two columns, *i.e.*, $\mathbf{g}_{\bullet 1} \mathrel{\mathring{=}}_{ij} \mathbf{g}_{\bullet 2}$). Hence,

$$\mathbf{G} = \begin{pmatrix} 1 & 0 & \mathbf{x}^\mathsf{T} \\ 0 & 1 & \mathbf{x}^\mathsf{T} \\ \mathbf{y} & \mathbf{y} & \mathbf{B} \end{pmatrix} \quad \text{and} \quad \mathbf{G}_{(-1)} = \begin{pmatrix} 1 & \mathbf{x}^\mathsf{T} \\ \mathbf{y} & \mathbf{B} \end{pmatrix},$$

with length $n-2$ vectors $\mathbf{x}, \mathbf{y} \geqq \mathbf{0}$, and an $(n-2) \times (n-2)$ matrix $\mathbf{B}$ satisfying Assumption 1. Let $(\mu, \mathbf{q})$ be the leading eigenpair (*i.e.*, the Perron root and the Perron eigenvector) of $\mathbf{G}$, then

$$\mathbf{0} = (\mathbf{G} - \mu\mathbf{I}) \cdot \mathbf{q} = \left(\begin{pmatrix} 1 & 0 & \mathbf{x}^\mathsf{T} \\ 0 & 1 & \mathbf{x}^\mathsf{T} \\ \mathbf{y} & \mathbf{y} & \mathbf{B} \end{pmatrix} - \mu\mathbf{I} \right) \cdot \begin{pmatrix} 1 \\ 1 \\ \mathbf{q}_{(-[2])} \end{pmatrix} = \begin{pmatrix} 1 - \mu + \mathbf{x}^\mathsf{T} \cdot \mathbf{q}_{(-[2])} \\ 1 - \mu + \mathbf{x}^\mathsf{T} \cdot \mathbf{q}_{(-[2])} \\ 2\mathbf{y} + (\mathbf{B} - \mu\mathbf{I})\mathbf{q}_{(-[2])} \end{pmatrix}.$$

Since the first two equations coincide, we may drop either of both, arriving at

$$\mathbf{0} = \left(\begin{pmatrix} 1 & \mathbf{x}^\mathsf{T} \\ 2\mathbf{y} & \mathbf{B} \end{pmatrix} - \mu\mathbf{I} \right) \cdot \begin{pmatrix} 1 \\ \mathbf{q}_{(-[2])} \end{pmatrix} = \left(\tilde{\mathbf{G}} - \mu\mathbf{I} \right) \cdot \mathbf{q}_{(-1)},$$

where

$$\tilde{\mathbf{G}} \equiv \begin{pmatrix} 1 & \mathbf{x}^\mathsf{T} \\ 2\mathbf{y} & \mathbf{B} \end{pmatrix} = \mathbf{G}_{(-1)} + \begin{pmatrix} 0 & \mathbf{0}^\mathsf{T} \\ \mathbf{y} & \mathbf{0} \end{pmatrix} = \mathbf{G}_{(-1)} + \mathbf{Y},$$

with $\mathbf{Y} \equiv \tilde{\mathbf{y}} \cdot \mathbf{e}_1^\mathsf{T}$ and $\tilde{\mathbf{y}} \equiv (0, \mathbf{y}^\mathsf{T})^\mathsf{T}$. We decompose the perturbation

$$\mathbf{Y} = \sum_{i=2}^{n} \mathbf{Y}_i, \quad \text{with} \quad \mathbf{Y}_i \equiv \tilde{y}_i \, \mathbf{e}_i \cdot \mathbf{e}_1^\mathsf{T}.$$

into $n-1$ successive perturbations $\mathbf{Y}_i$, $i = 2, \ldots, n$. Since perturbation $\mathbf{Y}_i$ (weakly) increases the ith row of $\mathbf{G}_{(-1)}$ leaving all other rows unaffected, we know from [90, Theorem 3.1] that this perturbation (weakly) increases the ith component of the (normalized) Perron vector of $\mathbf{G}_{(-1)}$. Successively repeating this argument, all $n-1$ components of the Perron vector are (weakly) increased relative to the first component. □
