# Supplementary material for: Eigenvalue productivity: Measurement of individual contributions in teams
Source: PLoS One. 2022 Sep 15;17(9):e0273623. doi: 10.1371/journal.pone.0273623 (PMC9477377; doi:10.1371/journal.pone.0273623)
Supplement: S2 Appendix — (PDF) [file pone.0273623.s002.pdf]

S2 Appendix. Example 2: Success of teams in movies.
The movie industry offers suitable data to exemplify the applicability of EVP. Since movies are produced by varying teams of directors, actors, directors of photography, writers etc., team compositions change from one movie to another. Apparently, for any given genre of movies, some teams perform better or are more successful than others, implying that the team composition is crucial for the specific success of a movie. At the same time, success measures for movies are readily available: numbers of moviegoers, license fees, awards received, rankings in journals or on internet platforms may be used as indicators of success or quality.

In order to illustrate that our concept of EVP also functions well on this data, we consider a particular class of movies and the team of actors and directors of photography producing them, and compute the EVP values for all of those team members. To keep the example tractable, and thus the number of movies manageable, we consider a class of movies where the castings have significant overlaps, so that pairs of team members repeatedly take part in different movies of this class. To this end, we restrict the class of movies to a specific class: movies directed by the German director, movie maker and actor Rainer Werner Fassbinder. (Fassbinder, born May 31, 1945 in Bad Wörishofen, Germany, died June 10, 1982 in Munich, Germany, is considered as one of the most important representatives of the so-called New German Cinema movement.) Among other works, his oeuvre includes 40 feature-length movies that he completed between 1969 and 1982. (In addition, he produced three short movies (*This Night*, *Der Stadtstreicher*, and *Das kleine Chaos*), an episode within the movie *Deutschland im Herbst*, and a TV–version of the cinema movie *Bolwieser*.) From those 40 movies we exclude *Theater in Trance*, because it is a documentary movie, and *Wie ein Vogel auf dem Draht*, because it is basically a one–woman TV show (for and of Brigitte Mira). Disregarding these movies, our data set contains 38 movies with 38 different teams. By construction, as a producer Fassbinder is a member of all teams, and thus we remove him unless he (also) has a role as an actor (9 movies). Our data set contains the main actors, including those who play a 'significant' part, and the directors of photography for those 38 movies—altogether 23 team members. The complete list of these 38 movies and 23 team members is included in Tables 4 and 5, respectively. The data was collected on the Homepage of the Rainer Werner Fassbinder Foundation [91]. We use the *IMDb*–user rating as an indicator of success of a movie ([92]).

Since we repeat the same steps as in Example 1, we only provide the basic results. First, we use the *IMDb*–ratings to compute *IMDb*–based scores for each artist, which are displayed in the fifth column of Table 5. Then, we compute the *IMDb*–based scores for each pair of artists, and use this data to compute the matrices $\mathbf{S}$ and $\mathbf{G}$, displayed on page 25. Finally, we use matrix $\mathbf{G}$ to compute the EVP–vector: We find that the (absolutely) largest eigenvalue is $\hat{\lambda} = 22.9679$, and the associated eigenvector is (after normalizing $\sum \mathbf{p}_i = n$)

$$\mathbf{p}(\hat{\lambda}) = (0.968, 0.995, 0.988, 0.979, 0.986, 1.048, 0.995, 0.943, 1.036, 1., 1.043, 1.021, 0.96, 1.04, 0.986, 1.001, 0.969, 0.985, 1.024, 0.977, 1.013, 1.01, 1.034).$$

The ranking of the artists implied by $\mathbf{p}(\hat{\lambda})$ is provided in Table 5. In order to check the plausibility of EVP, we compare $\mathbf{p}(\hat{\lambda})$ with the *IMDb*–score: As the coefficient of correlation between EVP and the *IMDb*–score is 0.9601, this suggests that EVP works quite well on that data, yielding plausible team productivities and thus a plausible ranking.

Table 4. Full-length Fassbinder movies: teams and *IMDb*–user ratings

no.	team[a]	*IMDb*	German title	English title	year
4.	*EGJOQT*	6.7	*Liebe ist kälter als der Tod*	*Love is Colder than Death*	1969
5.	*ADHOQRT*	6.5	*Götter der Pest*	*Gods of the Plague*	1969
6.	*ABEFGOQT*	7.2	*Katzelmacher*	*Katzelmacher*	1969
7.	*AKLQT*	7.7	*Warum läuft Herr R. Amok?*	*Why does Herr R. run Amok?*	1969
8.	*HMORT*	6.5	*Rio das Mortes*	*Rio das Mortes*	1979
9.	*ABCDGHLO*	6.6	*Das Kaffeehaus*	*Das Kaffeehaus*	1970
10.	*AHJMOQRU*	6.6	*Whity*	*Whity*	1970
11.	*CEOQT*	6.1	*Niklashauser Fahrt*	*The Niklashausen Journey*	1970
12.	*EMNRT*	6.8	*Der amerikanische Soldat*	*The American Soldier*	1970
13.	*DEJLNOQU*	7.0	*Warnung vor einer heiligen Nutte*	*Beware of a Holy Whore*	1970
14.	*ABFHORT*	6.6	*Pioniere in Ingolstadt*	*Pioneers in Ingolstadt*	1970
15.	*FGINRT*	7.6	*Händler der vier Jahreszeiten*	*The Merchant of Four Seasons*	1971
16.	*CFMORU*	7.8	*Die bitteren Tränen der Petra von Kant*	*The Bitter Tears of Petra von Kant*	1971
17.	*ABRT*	7.2	*Wildwechsel*	*Jailbait*	1972
18.	*BFGJLNOT*	8.5	*Acht Stunden sind kein Tag*	*Eight Hours are not a Day*	1972
19.	*ACEJOT*	7.6	*Bremer Freiheit*	*Bremen Freedom*	1972
20.	*CDIJKLNU*	7.9	*Welt am Draht*	*World on a Wire*	1973
21.	*CFIJK*	7.2	*Nora Helmer*	*Nora Helmer*	1973
22.	*HIU*	7.8	*Martha*	*Martha*	1973
23.	*EFKNRV*	8.1	*Angst essen Seele auf*	*Fear Eats the Soul*	1973
24.	*FJKNORTV*	7.1	*Fontane Effi Briest*	*Effi Briest*	1974
25.	*AEFLNRU*	7.8	*Faustrecht der Freiheit*	*Fox and his Friends*	1974
27.	*CDFKLRU*	7.7	*Mutter Küsters' Fahrt zum Himmel*	*Mother Küster Goes to Heaven*	1975
28.	*CDFKLV*	7.7	*Angst vor der Angst*	*Fear of Fear*	1975
29.	*U*	8.0	*Ich will doch nur, dass ihr mich liebt*	*I Only Want You to Love Me*	1975
30.	*CDJLPRUV*	7.2	*Satansbraten*	*Satan's Brew*	1975
31.	*CJPSU*	7.6	*Chinesisches Roulette*	*Chinese Roulette*	1976
33.	*LPQSU*	7.3	*Bolwieser (Kino)*	*The Stationmaster's Wife*	1976
34.	*CU*	7.5	*Frauen in New York*	*Women in New York*	1977
35.	*IPQSU*	7.4	*Despair — Eine Reise ins Licht*	*Despair*	1977
37.	*IKOQSU*	7.9	*Die Ehe der Maria Braun*	*The Marriage of Maria Braun*	1978
38.	*DHKNP*	7.7	*In einem Jahr mit 13 Monden*	*I a Year of 13 Moons*	1978
39.	*ACHOPS*	7.1	*Die dritte Generation*	*The Third Generation*	1978
40.	*FKOQSW*	8.8	*Berlin Alexanderplatz*	*Berlin Alexanderplatz*	1979
41.	*AEKOQSW*	7.2	*Lili Marleen*	*Lili Marleen*	1980
42.	*QSW*	7.7	*Lola*	*Lola*	1981
44.	*SW*	8.0	*Die Sehnsucht der Veronika Voss*	*Veronika Vross*	1981
45.	*HQSW*	6.9	*Querelle*	*Querelle*	1981

[a]Encoding of team members according to labels in Table 5.

Table 5. EVP–ranking of the artist

rank	artist	label	no. movies	*IMDb*	EVP
1	Irm Herrmann	*F*	12	0.768	1.048
2	Lilo Pempeit	*K*	11	0.773	1.043
3	Karl Scheydt	*N*	9	0.761	1.04
4	Klaus Löwitsch	*I*	6	0.763	1.036
5	Xavier Schwarzenberger	*W*	5	0.772	1.034
6	Juliane Lorenz	*S*	10	0.759	1.024
7	Kurt Raab	*L*	10	0.754	1.021
8	Michael Ballhaus	*U*	14	0.754	1.013
9	Jürgen Jürgens	*V*	4	0.752	1.01
10	Volker Sprengler	*P*	6	0.738	1.001
11	Ulli Lommel	*J*	10	0.734	1.
12	Rudolf Waldemar Brehm	*B*	5	0.722	0.995
13	Hans Hirschmüller	*G*	5	0.732	0.995
14	Margit Carstensen	*C*	12	0.733	0.988
15	Hannah Schygulla	*O*	17	0.716	0.986
16	Rainer Werner Fassbinder	*E*	9	0.717	0.986
17	Thea Eymesz	*R*	13	0.719	0.985
18	Ingrid Caven	*D*	8	0.729	0.979
19	Dietrich Lohman	*T*	13	0.708	0.977
20	Franz Walsch	*Q*	14	0.721	0.969
21	Harry Baer	*A*	11	0.71	0.968
22	Katrin Schaake	*M*	4	0.693	0.96
23	Günther Kaufmann	*H*	9	0.692	0.943

$$
\mathbf{S} = \begin{pmatrix}
0.71 & 0.69 & 0.71 & 0.655 & 0.745 & 0.72 & 0.69 & 0.668 & 0.736 & 0.71 & 0.745 & 0.737 & 0.66 & 0.78 & 0.692 & 0.71 & 0.704 & 0.694 & 0.715 & 0.713 & 0.72 & 0.731 & 0.72 \\
0.69 & 0.722 & 0.66 & 0.66 & 0.72 & 0.743 & 0.743 & 0.66 & 0.742 & 0.85 & 0.747 & 0.755 & 0.707 & 0.85 & 0.722 & 0.73 & 0.72 & 0.69 & 0.74 & 0.737 & 0.738 & 0.737 & 0.747 \\
0.71 & 0.66 & 0.733 & 0.742 & 0.685 & 0.76 & 0.66 & 0.685 & 0.755 & 0.75 & 0.762 & 0.742 & 0.78 & 0.79 & 0.704 & 0.73 & 0.61 & 0.757 & 0.735 & 0.685 & 0.762 & 0.745 & 0.752 \\
0.655 & 0.66 & 0.742 & 0.729 & 0.7 & 0.77 & 0.66 & 0.693 & 0.79 & 0.737 & 0.775 & 0.735 & 0.71 & 0.753 & 0.67 & 0.745 & 0.675 & 0.713 & 0.744 & 0.65 & 0.745 & 0.745 & 0.75 \\
0.745 & 0.72 & 0.685 & 0.7 & 0.717 & 0.77 & 0.695 & 0.704 & 0.74 & 0.71 & 0.765 & 0.74 & 0.68 & 0.742 & 0.697 & 0.727 & 0.684 & 0.757 & 0.72 & 0.688 & 0.74 & 0.81 & 0.72 \\
0.72 & 0.743 & 0.76 & 0.77 & 0.77 & 0.768 & 0.777 & 0.66 & 0.74 & 0.76 & 0.777 & 0.793 & 0.78 & 0.782 & 0.767 & 0.753 & 0.8 & 0.753 & 0.88 & 0.74 & 0.777 & 0.763 & 0.88 \\
0.69 & 0.743 & 0.66 & 0.66 & 0.695 & 0.777 & 0.732 & 0.66 & 0.76 & 0.76 & 0.752 & 0.755 & 0.712 & 0.805 & 0.725 & 0.735 & 0.695 & 0.76 & 0.745 & 0.75 & 0.743 & 0.742 & 0.752 \\
0.668 & 0.66 & 0.685 & 0.693 & 0.704 & 0.66 & 0.66 & 0.692 & 0.78 & 0.66 & 0.77 & 0.66 & 0.655 & 0.77 & 0.665 & 0.74 & 0.667 & 0.655 & 0.7 & 0.653 & 0.72 & 0.722 & 0.69 \\
0.736 & 0.742 & 0.755 & 0.79 & 0.74 & 0.74 & 0.76 & 0.78 & 0.763 & 0.755 & 0.767 & 0.79 & 0.727 & 0.775 & 0.79 & 0.74 & 0.765 & 0.76 & 0.765 & 0.76 & 0.775 & 0.758 & 0.768 \\
0.71 & 0.85 & 0.75 & 0.737 & 0.71 & 0.76 & 0.76 & 0.66 & 0.755 & 0.734 & 0.74 & 0.765 & 0.66 & 0.762 & 0.725 & 0.74 & 0.677 & 0.697 & 0.76 & 0.748 & 0.726 & 0.715 & 0.753 \\
0.745 & 0.747 & 0.762 & 0.775 & 0.765 & 0.777 & 0.752 & 0.77 & 0.767 & 0.74 & 0.773 & 0.775 & 0.732 & 0.77 & 0.775 & 0.77 & 0.79 & 0.763 & 0.797 & 0.74 & 0.783 & 0.763 & 0.8 \\
0.737 & 0.755 & 0.742 & 0.735 & 0.74 & 0.793 & 0.755 & 0.66 & 0.79 & 0.765 & 0.775 & 0.754 & 0.723 & 0.78 & 0.737 & 0.725 & 0.733 & 0.757 & 0.73 & 0.81 & 0.748 & 0.745 & 0.763 \\
0.66 & 0.707 & 0.78 & 0.71 & 0.68 & 0.78 & 0.712 & 0.655 & 0.727 & 0.66 & 0.732 & 0.723 & 0.693 & 0.68 & 0.697 & 0.715 & 0.66 & 0.692 & 0.725 & 0.665 & 0.72 & 0.722 & 0.731 \\
0.78 & 0.85 & 0.79 & 0.753 & 0.742 & 0.782 & 0.805 & 0.77 & 0.775 & 0.762 & 0.77 & 0.78 & 0.68 & 0.761 & 0.753 & 0.77 & 0.7 & 0.748 & 0.76 & 0.75 & 0.757 & 0.76 & 0.767 \\
0.692 & 0.722 & 0.704 & 0.67 & 0.697 & 0.767 & 0.725 & 0.665 & 0.79 & 0.725 & 0.775 & 0.737 & 0.697 & 0.753 & 0.716 & 0.71 & 0.711 & 0.685 & 0.775 & 0.698 & 0.732 & 0.71 & 0.8 \\
0.71 & 0.73 & 0.73 & 0.745 & 0.727 & 0.753 & 0.735 & 0.74 & 0.74 & 0.74 & 0.77 & 0.725 & 0.715 & 0.77 & 0.71 & 0.738 & 0.735 & 0.72 & 0.735 & 0.723 & 0.738 & 0.72 & 0.755 \\
0.704 & 0.72 & 0.61 & 0.675 & 0.684 & 0.8 & 0.695 & 0.667 & 0.765 & 0.677 & 0.79 & 0.733 & 0.66 & 0.7 & 0.711 & 0.735 & 0.721 & 0.655 & 0.76 & 0.684 & 0.724 & 0.737 & 0.765 \\
0.694 & 0.69 & 0.757 & 0.713 & 0.757 & 0.753 & 0.76 & 0.655 & 0.76 & 0.697 & 0.763 & 0.757 & 0.692 & 0.748 & 0.685 & 0.72 & 0.655 & 0.719 & 0.739 & 0.69 & 0.742 & 0.747 & 0.745 \\
0.715 & 0.74 & 0.735 & 0.744 & 0.72 & 0.88 & 0.745 & 0.7 & 0.765 & 0.76 & 0.797 & 0.73 & 0.725 & 0.76 & 0.775 & 0.735 & 0.76 & 0.739 & 0.759 & 0.733 & 0.755 & 0.756 & 0.772 \\
0.713 & 0.737 & 0.685 & 0.65 & 0.688 & 0.74 & 0.75 & 0.653 & 0.76 & 0.748 & 0.74 & 0.81 & 0.665 & 0.75 & 0.698 & 0.723 & 0.684 & 0.69 & 0.733 & 0.708 & 0.731 & 0.71 & 0.74 \\
0.72 & 0.738 & 0.762 & 0.745 & 0.74 & 0.777 & 0.743 & 0.72 & 0.775 & 0.726 & 0.783 & 0.748 & 0.72 & 0.757 & 0.732 & 0.738 & 0.724 & 0.742 & 0.755 & 0.731 & 0.754 & 0.72 & 0.763 \\
0.731 & 0.737 & 0.745 & 0.745 & 0.81 & 0.763 & 0.742 & 0.722 & 0.758 & 0.715 & 0.763 & 0.745 & 0.722 & 0.76 & 0.71 & 0.72 & 0.737 & 0.747 & 0.756 & 0.71 & 0.72 & 0.752 & 0.762 \\
0.72 & 0.747 & 0.752 & 0.75 & 0.72 & 0.88 & 0.752 & 0.69 & 0.768 & 0.753 & 0.8 & 0.763 & 0.731 & 0.767 & 0.8 & 0.755 & 0.765 & 0.745 & 0.772 & 0.74 & 0.763 & 0.762 & 0.772
\end{pmatrix}
$$

$$
\mathbf{G} = \begin{pmatrix}
1. & 0.956 & 0.968 & 0.899 & 1.04 & 0.938 & 0.943 & 0.965 & 0.964 & 0.967 & 0.964 & 0.977 & 0.953 & 1.025 & 0.967 & 0.962 & 0.976 & 0.965 & 0.942 & 1.007 & 0.955 & 0.971 & 0.933 \\
0.972 & 1. & 0.9 & 0.906 & 1.005 & 0.969 & 1.015 & 0.953 & 0.973 & 1.158 & 0.967 & 1.001 & 1.021 & 1.117 & 1.008 & 0.989 & 0.998 & 0.959 & 0.975 & 1.041 & 0.979 & 0.98 & 0.967 \\
1. & 0.914 & 1. & 1.018 & 0.956 & 0.99 & 0.902 & 0.99 & 0.989 & 1.022 & 0.987 & 0.984 & 1.126 & 1.038 & 0.983 & 0.989 & 0.846 & 1.052 & 0.968 & 0.967 & 1.011 & 0.99 & 0.975 \\
0.923 & 0.914 & 1.012 & 1. & 0.977 & 1.003 & 0.902 & 1.002 & 1.035 & 1.004 & 1.003 & 0.975 & 1.026 & 0.99 & 0.935 & 1.009 & 0.936 & 0.992 & 0.98 & 0.917 & 0.989 & 0.99 & 0.972 \\
1.049 & 0.997 & 0.934 & 0.961 & 1. & 1.003 & 0.949 & 1.018 & 0.969 & 0.967 & 0.99 & 0.981 & 0.982 & 0.976 & 0.972 & 0.985 & 0.948 & 1.052 & 0.949 & 0.971 & 0.982 & 1.076 & 0.933 \\
1.014 & 1.03 & 1.036 & 1.057 & 1.074 & 1. & 1.061 & 0.953 & 0.969 & 1.035 & 1.005 & 1.051 & 1.126 & 1.027 & 1.07 & 1.02 & 1.109 & 1.047 & 1.159 & 1.045 & 1.031 & 1.014 & 1.14 \\
0.972 & 1.03 & 0.9 & 0.906 & 0.97 & 1.012 & 1. & 0.953 & 0.996 & 1.035 & 0.973 & 1.001 & 1.028 & 1.058 & 1.012 & 0.996 & 0.963 & 1.057 & 0.982 & 1.059 & 0.986 & 0.986 & 0.974 \\
0.941 & 0.914 & 0.934 & 0.951 & 0.983 & 0.86 & 0.902 & 1. & 1.022 & 0.899 & 0.996 & 0.875 & 0.946 & 1.012 & 0.928 & 1.002 & 0.924 & 0.911 & 0.922 & 0.922 & 0.955 & 0.959 & 0.894 \\
1.037 & 1.028 & 1.03 & 1.084 & 1.032 & 0.964 & 1.038 & 1.127 & 1. & 1.029 & 0.992 & 1.048 & 1.05 & 1.018 & 1.103 & 1.002 & 1.06 & 1.057 & 1.008 & 1.073 & 1.028 & 1.007 & 0.994 \\
1. & 1.177 & 1.023 & 1.011 & 0.991 & 0.99 & 1.038 & 0.953 & 0.989 & 1. & 0.958 & 1.015 & 0.953 & 1.002 & 1.012 & 1.002 & 0.938 & 0.969 & 1.001 & 1.055 & 0.963 & 0.95 & 0.975 \\
1.049 & 1.035 & 1.04 & 1.063 & 1.067 & 1.012 & 1.027 & 1.112 & 1.004 & 1.008 & 1. & 1.028 & 1.056 & 1.012 & 1.082 & 1.043 & 1.095 & 1.061 & 1.05 & 1.045 & 1.039 & 1.014 & 1.036 \\
1.038 & 1.046 & 1.012 & 1.009 & 1.033 & 1.033 & 1.031 & 0.953 & 1.035 & 1.042 & 1.003 & 1. & 1.043 & 1.025 & 1.028 & 0.982 & 1.017 & 1.052 & 0.962 & 1.143 & 0.993 & 0.99 & 0.988 \\
0.93 & 0.979 & 1.064 & 0.975 & 0.949 & 1.016 & 0.973 & 0.946 & 0.952 & 0.899 & 0.947 & 0.958 & 1. & 0.893 & 0.972 & 0.968 & 0.915 & 0.963 & 0.955 & 0.939 & 0.955 & 0.959 & 0.947 \\
1.099 & 1.177 & 1.077 & 1.034 & 1.036 & 1.019 & 1.1 & 1.112 & 1.015 & 1.039 & 0.996 & 1.034 & 0.982 & 1. & 1.051 & 1.043 & 0.97 & 1.04 & 1.001 & 1.059 & 1.004 & 1.01 & 0.993 \\
0.975 & 1.001 & 0.96 & 0.919 & 0.972 & 0.999 & 0.99 & 0.961 & 1.035 & 0.988 & 1.003 & 0.977 & 1.006 & 0.99 & 1. & 0.962 & 0.986 & 0.952 & 1.021 & 0.985 & 0.972 & 0.944 & 1.036 \\
1. & 1.011 & 0.995 & 1.022 & 1.015 & 0.981 & 1.004 & 1.069 & 0.969 & 1.008 & 0.996 & 0.962 & 1.033 & 1.012 & 0.991 & 1. & 1.019 & 1.001 & 0.968 & 1.021 & 0.979 & 0.957 & 0.978 \\
0.992 & 0.997 & 0.832 & 0.926 & 0.954 & 1.042 & 0.949 & 0.963 & 1.002 & 0.922 & 1.022 & 0.973 & 0.953 & 0.92 & 0.993 & 0.995 & 1. & 0.911 & 1.001 & 0.965 & 0.961 & 0.979 & 0.991 \\
0.977 & 0.956 & 1.032 & 0.979 & 1.056 & 0.981 & 1.038 & 0.946 & 0.996 & 0.949 & 0.988 & 1.004 & 1. & 0.983 & 0.956 & 0.975 & 0.908 & 1. & 0.973 & 0.974 & 0.985 & 0.992 & 0.965 \\
1.007 & 1.025 & 1.002 & 1.021 & 1.005 & 1.147 & 1.018 & 1.011 & 1.002 & 1.035 & 1.031 & 0.968 & 1.047 & 0.999 & 1.082 & 0.995 & 1.053 & 1.027 & 1. & 1.035 & 1.002 & 1.004 & 1. \\
1.005 & 1.021 & 0.934 & 0.892 & 0.96 & 0.964 & 1.025 & 0.944 & 0.996 & 1.018 & 0.958 & 1.074 & 0.96 & 0.985 & 0.974 & 0.98 & 0.948 & 0.959 & 0.966 & 1. & 0.97 & 0.944 & 0.958 \\
1.014 & 1.022 & 1.039 & 1.022 & 1.033 & 1.012 & 1.015 & 1.04 & 1.015 & 0.989 & 1.014 & 0.992 & 1.04 & 0.994 & 1.022 & 0.999 & 1.004 & 1.032 & 0.995 & 1.031 & 1. & 0.957 & 0.988 \\
1.029 & 1.021 & 1.016 & 1.022 & 1.13 & 0.995 & 1.014 & 1.043 & 0.993 & 0.974 & 0.988 & 0.988 & 1.042 & 0.999 & 0.991 & 0.975 & 1.021 & 1.038 & 0.996 & 1.002 & 0.955 & 1. & 0.987 \\
1.014 & 1.034 & 1.026 & 1.029 & 1.005 & 1.147 & 1.027 & 0.997 & 1.006 & 1.026 & 1.035 & 1.012 & 1.056 & 1.007 & 1.117 & 1.023 & 1.06 & 1.036 & 1.017 & 1.044 & 1.012 & 1.013 & 1.
\end{pmatrix}
$$
