# Supplementary material for: Eigenvalue productivity: Measurement of individual contributions in teams
Source: PLoS One. 2022 Sep 15;17(9):e0273623. doi: 10.1371/journal.pone.0273623 (PMC9477377; doi:10.1371/journal.pone.0273623)
Supplement: S3 Appendix — (PDF) [file pone.0273623.s003.pdf]

S3 Appendix. Example 3: A research group.
With the advance of multiple coauthors of scientific publications, the question how to allocate the credit of a publication to different coauthors becomes a widely debated issue (see, for example, [93–96]). We here show how EVP may be used to allocate the credit of multi–author publications.

Consider a team consisting of a group of researchers of various economics departments at European universities and take their publication records as a measure of the success of cooperation within this team. (The data source is an existing research group, but to guarantee anonymity we do not use real names. The details of the publication records were collected from the researchers' personal and/or institutional websites.) As we are interested in the mutual productivity effects between the members of this group and their implied pairwise productivity coefficients, we disregard any stand-alone work as well as joint work with external, *i.e.*, out of group researchers. Hence, we drop all external coauthors and, after their removal, also disregard all single-authored publications. This procedure helps focus on the immediate cooperation of the members of the research group and their productivity effects; also, it avoids boosting the example excessively by including a large number of external researchers.

After removing those publications that are not coauthored by at least two members of the research group, the team consists of 14 members labeled by

$$\mathcal{N} = \{A, B, C, F, G, H, I, J, K, L, M, N, O, P\}.$$

The members of team $\mathcal{N}$ have published 45 articles in peer-reviewed journals within the two-year period 2015–2016. They collaborated in 26 different teams of coauthors, publishing up to seven research articles a team. Each member of $\mathcal{N}$ collaborated in up to 13 different teams of coauthors, publishing up to 15 research articles (team member C).

To measure the significance of a publication (success of a coauthorship) we use the ranking of economics journals established by the German economic newspaper *Handelsblatt* (*HB*) in 2015, [97]. This list assigns each (established) journal to either of the categories: `A+`, `A`, `B+`, `B`, `C+`, `C`, and `D`, which are associated with the cardinal measures (points) 1, 0.6, 0.3, 0.2, 0.15, 0.1 and 0.05, respectively. We multiply these points with 100 such that the maximal possible points for a publication are 100 for a publication in a top journal (`A+`), and 5 for a mediocre journal (`D`). Publications in journals that were not included in the *HB* ranking are credited with 5 points.

Table 6 represents our database consisting of the publication data associated with the joint publications of the team members. The columns of this table display the (internal) coauthors, the number of publications of this team of coauthors (frequency with which the team of authors appears in our data set), the number of all coauthors (internal and external), the number of internal coauthors (members of $\mathcal{N}$) and the ranking score of the journal. The average points achieved by a paper amounts to 22.67, while the average points achieved by a team of coauthors amounts to 22.02, both of

Table 6. Results of the research group

no.	team of coauthors	no. publications	no. authors	no. authors internal	journal ranking
1	*ACK*	1	5	3	100
2	*AO*	7	7	2	10
3	*BCK*	1	4	3	20
4	*CFK*	1	4	3	10
5	*AMO*	3	4	3	60
6	*AO*	7	4	2	5
7	*AMO*	3	4	3	60
8	*BM*	1	2	2	5
9	*BC*	2	2	2	20
10	*BC*	2	3	2	5
11	*CJMN*	1	4	4	20
12	*CH*	1	3	2	20
13	*CHIK*	1	5	4	5
14	*HO*	3	5	2	10
15	*HO*	3	4	2	5
16	*CK*	1	2	2	60
17	*BCFM*	1	5	4	60
18	*JK*	1	5	2	5
19	*JP*	4	3	2	60
20	*JLP*	2	4	3	5
21	*BJ*	2	3	2	10
22	*JP*	4	4	2	30
23	*JLP*	2	3	3	5
24	*KN*	1	13	2	5
25	*IK*	3	3	2	5
26	*IK*	3	3	2	20
27	*FK*	1	3	2	10
28	*CGK*	1	4	3	5
29	*CHKL*	1	4	4	5
30	*IK*	3	2	2	20
31	*AO*	7	2	2	30
32	*AO*	7	3	2	20
33	*AMO*	3	3	3	60
34	*AO*	7	2	2	15
35	*AO*	7	5	2	30
36	*BJ*	2	3	2	5
37	*CMN*	2	3	3	20
38	*CN*	1	3	2	10
39	*CMN*	2	4	3	60
40	*MN*	1	2	2	10
41	*HO*	3	4	2	5
42	*AO*	7	4	2	10
43	*JP*	4	2	2	5
44	*JP*	4	3	2	60
45	*KP*	1	4	2	20

which is slightly better than a publication in a B–journal. Using the data set shown in Table 6, we compile the data for any particular team; this data is provided in Table 7.

Table 7. Publications of the different coauthorships

coauthors	publications	no. papers	points	max points	success ratio	
ACK	$\{1\}$	1	100	100	1	1.
AMO	$\{5, 7, 33\}$	3	180	300	$\frac{3}{5}$	0.6
AO	$\{2, 6, 31, 32, 34, 35, 42\}$	7	120	700	$\frac{6}{35}$	0.171
BC	$\{9, 10\}$	2	25	200	$\frac{1}{8}$	0.125
$BCFM$	$\{17\}$	1	60	100	$\frac{3}{5}$	0.6
BCK	$\{3\}$	1	20	100	$\frac{1}{5}$	0.2
BJ	$\{21, 36\}$	2	15	200	$\frac{3}{40}$	0.075
BM	$\{8\}$	1	5	100	$\frac{1}{20}$	0.05
CFK	$\{4\}$	1	10	100	$\frac{1}{10}$	0.1
CGK	$\{28\}$	1	5	100	$\frac{1}{20}$	0.05
CH	$\{12\}$	1	20	100	$\frac{1}{5}$	0.2
$CHIK$	$\{13\}$	1	5	100	$\frac{1}{20}$	0.05
$CHKL$	$\{29\}$	1	5	100	$\frac{1}{20}$	0.05
$CJMN$	$\{11\}$	1	20	100	$\frac{1}{5}$	0.2
CK	$\{16\}$	1	60	100	$\frac{3}{5}$	0.6
CMN	$\{37, 39\}$	2	80	200	$\frac{2}{5}$	0.4
CN	$\{38\}$	1	10	100	$\frac{1}{10}$	0.1
FK	$\{27\}$	1	10	100	$\frac{1}{10}$	0.1
HO	$\{14, 15, 41\}$	3	20	300	$\frac{1}{15}$	0.067
IK	$\{25, 26, 30\}$	3	45	300	$\frac{3}{20}$	0.15
JK	$\{18\}$	1	5	100	$\frac{1}{20}$	0.05
JLP	$\{20, 23\}$	2	10	200	$\frac{1}{20}$	0.05
JP	$\{19, 22, 43, 44\}$	4	155	400	$\frac{31}{80}$	0.388
KN	$\{24\}$	1	5	100	$\frac{1}{20}$	0.05
KP	$\{45\}$	1	20	100	$\frac{1}{5}$	0.2
MN	$\{40\}$	1	10	100	$\frac{1}{10}$	0.1

From Table 6 we calculate the results for each single researcher $i \in \mathcal{N}$ by focusing on those projects where i was included in the team of authors. The individual results are shown in Table 8. The individual success ratio s_i represents the individual HB–score. While this score measures the success of each member of the team accomplished in collaboration with different sets of coauthors, it disregards the specific team compositions and hence the coworker productivities. Acknowledging these coworker productivities, EVP will (generically) result in a measure of coworker productivity different from the HB–score; however, we would, of course, expect a strong (positive) correlation between both, the HB–score and EVP. (We use the

HB–scores to compare the EVP values with some established ranking method; yet, any other reasonable ranking method may equally serve to check the plausibility of EVP.)

Table 8. Publication success of the team members

author	no. teams	no. publ.	points	success ratio s_i	
A	3	11	400	$\frac{4}{11}$	0.364
B	5	7	125	$\frac{5}{28}$	0.179
C	13	15	420	$\frac{7}{25}$	0.28
F	3	3	80	$\frac{4}{15}$	0.267
G	1	1	5	$\frac{1}{20}$	0.05
H	4	6	50	$\frac{1}{12}$	0.083
I	2	4	50	$\frac{1}{8}$	0.125
J	5	10	205	$\frac{41}{200}$	0.205
K	12	14	290	$\frac{29}{140}$	0.207
L	2	3	15	$\frac{1}{20}$	0.05
M	6	9	355	$\frac{71}{180}$	0.394
N	5	6	125	$\frac{5}{24}$	0.208
O	3	13	320	$\frac{16}{65}$	0.246
P	3	7	185	$\frac{37}{140}$	0.264

In the next step, we calculate the success ratio for each pair of researchers $\{i, j\}, i, j \in \mathcal{N}$, which is done in a way analogous to that of calculating Table 8. For example, the pair $\{C, F\}$ collaborated in the teams $BCFM$ and CFK publishing two articles with scores 60 and 10; thus, their pair score equals $s_{CF} = 70/200 = 7/20$. Using that data, the matrix of pairwise success coefficients $\mathbf{S}$ is given as in Eq. (6). In the next step we use the matrix $\mathbf{S}$ and proceed to calculate the entries of $\mathbf{G}$ as described above (see page 16). The result is displayed in Eq. (7).

$$\mathbf{S} = \begin{pmatrix}
0.3636 & 0.2548 & 1. & 0.3114 & 0.1348 & 0.1741 & 0.2132 & 0.273 & 1. & 0.1348 & 0.6 & 0.2752 & 0.3 & 0.31 \\
0.2548 & 0.1786 & 0.2625 & 0.6 & 0.0945 & 0.122 & 0.1494 & 0.075 & 0.2 & 0.0945 & 0.325 & 0.1929 & 0.2097 & 0.2172 \\
1. & 0.2625 & 0.28 & 0.35 & 0.05 & 0.1 & 0.05 & 0.2 & 0.2929 & 0.05 & 0.4 & 0.275 & 0.2625 & 0.272 \\
0.3114 & 0.6 & 0.35 & 0.2667 & 0.1155 & 0.1491 & 0.1826 & 0.2338 & 0.1 & 0.1155 & 0.6 & 0.2357 & 0.2562 & 0.2655 \\
0.1348 & 0.0945 & 0.05 & 0.1155 & 0.05 & 0.0645 & 0.0791 & 0.1012 & 0.05 & 0.05 & 0.1404 & 0.1021 & 0.1109 & 0.115 \\
0.1741 & 0.122 & 0.1 & 0.1491 & 0.0645 & 0.0833 & 0.05 & 0.1307 & 0.05 & 0.05 & 0.1813 & 0.1318 & 0.0667 & 0.1484 \\
0.2132 & 0.1494 & 0.05 & 0.1826 & 0.0791 & 0.05 & 0.125 & 0.1601 & 0.125 & 0.0791 & 0.222 & 0.1614 & 0.1754 & 0.1818 \\
0.273 & 0.075 & 0.2 & 0.2338 & 0.1012 & 0.1307 & 0.1601 & 0.205 & 0.05 & 0.05 & 0.2 & 0.2 & 0.2246 & 0.275 \\
1. & 0.2 & 0.2929 & 0.1 & 0.05 & 0.05 & 0.125 & 0.05 & 0.2071 & 0.05 & 0.2858 & 0.05 & 0.2258 & 0.2 \\
0.1348 & 0.0945 & 0.05 & 0.1155 & 0.05 & 0.05 & 0.0791 & 0.05 & 0.05 & 0.05 & 0.1404 & 0.1021 & 0.1109 & 0.05 \\
0.6 & 0.325 & 0.4 & 0.6 & 0.1404 & 0.1813 & 0.222 & 0.2 & 0.2858 & 0.1404 & 0.3944 & 0.275 & 0.6 & 0.3229 \\
0.2752 & 0.1929 & 0.275 & 0.2357 & 0.1021 & 0.1318 & 0.1614 & 0.2 & 0.05 & 0.1021 & 0.275 & 0.2083 & 0.2265 & 0.2346 \\
0.3 & 0.2097 & 0.2625 & 0.2562 & 0.1109 & 0.0667 & 0.1754 & 0.2246 & 0.2258 & 0.1109 & 0.6 & 0.2265 & 0.2462 & 0.2551 \\
0.31 & 0.2172 & 0.272 & 0.2655 & 0.115 & 0.1484 & 0.1818 & 0.275 & 0.2 & 0.05 & 0.3229 & 0.2346 & 0.2551 & 0.2643
\end{pmatrix} \qquad (6)$$

$$\mathbf{G} = \begin{pmatrix}
1. & 1.427 & 3.5714 & 1.1677 & 2.6968 & 2.0889 & 1.7056 & 1.3319 & 4.8276 & 2.6968 & 1.5211 & 1.3212 & 1.2188 & 1.173 \\
0.7008 & 1. & 0.9375 & 2.25 & 1.8898 & 1.4639 & 1.1952 & 0.3659 & 0.9655 & 1.8898 & 0.8239 & 0.9258 & 0.8517 & 0.822 \\
2.75 & 1.47 & 1. & 1.3125 & 1. & 1.2 & 0.4 & 0.9756 & 1.4138 & 1. & 1.0141 & 1.32 & 1.0665 & 1.0293 \\
0.8563 & 3.36 & 1.25 & 1. & 2.3094 & 1.7889 & 1.4606 & 1.1405 & 0.4828 & 2.3094 & 1.5211 & 1.1314 & 1.0408 & 1.0045 \\
0.3708 & 0.5292 & 0.1786 & 0.433 & 1. & 0.7746 & 0.6325 & 0.4939 & 0.2414 & 1. & 0.356 & 0.4899 & 0.4507 & 0.435 \\
0.4787 & 0.6831 & 0.3571 & 0.559 & 1.291 & 1. & 0.4 & 0.6376 & 0.2414 & 1. & 0.4596 & 0.6325 & 0.2708 & 0.5615 \\
0.5863 & 0.8367 & 0.1786 & 0.6847 & 1.5811 & 0.6 & 1. & 0.7809 & 0.6034 & 1.5811 & 0.5629 & 0.7746 & 0.7126 & 0.6877 \\
0.7508 & 0.42 & 0.7143 & 0.8768 & 2.0248 & 1.5684 & 1.2806 & 1. & 0.2414 & 1. & 0.507 & 0.96 & 0.9126 & 1.0405 \\
2.75 & 1.12 & 1.0459 & 0.375 & 1. & 0.6 & 1. & 0.2439 & 1. & 1. & 0.7247 & 0.24 & 0.9173 & 0.7568 \\
0.3708 & 0.5292 & 0.1786 & 0.433 & 1. & 0.6 & 0.6325 & 0.2439 & 0.2414 & 1. & 0.356 & 0.4899 & 0.4507 & 0.1892 \\
1.65 & 1.82 & 1.4286 & 2.25 & 2.8087 & 2.1756 & 1.7764 & 0.9756 & 1.3799 & 2.8087 & 1. & 1.32 & 2.4375 & 1.2217 \\
0.7569 & 1.0801 & 0.9821 & 0.8839 & 2.0412 & 1.5811 & 1.291 & 0.9756 & 0.2414 & 2.0412 & 0.6972 & 1. & 0.92 & 0.8879 \\
0.825 & 1.1741 & 0.9376 & 0.9608 & 2.2188 & 0.8 & 1.4033 & 1.0958 & 1.0901 & 2.2188 & 1.5211 & 1.087 & 1. & 0.9651 \\
0.8525 & 1.2166 & 0.9715 & 0.9955 & 2.2991 & 1.7809 & 1.4541 & 1.3415 & 0.9655 & 1. & 0.8185 & 1.1263 & 1.0362 & 1.
\end{pmatrix} \qquad (7)$$

Table 9. EVP–ranking of the researchers

rank	author	EVP	HB–score	no. publ.
1	A	1.8082	0.3636	11
2	M	1.6208	0.3944	9
3	F	1.3146	0.2667	3
4	C	1.2889	0.28	15
5	O	1.1088	0.2462	13
6	P	1.0622	0.2643	7
7	B	1.033	0.1786	7
8	K	0.9855	0.2071	14
9	N	0.9282	0.2083	6
10	J	0.8132	0.205	10
11	I	0.6768	0.125	4
12	H	0.5212	0.0833	6
13	G	0.4386	0.05	1
14	L	0.4001	0.05	3

Finally, we have to calculate the eigenvalues of $\mathbf{G}$. Computing these, we find that the (absolutely) largest eigenvalue is $\hat{\lambda} = 14.4093$, and the associated eigenvector—the EVP–vector—is (after normalizing $\sum \mathbf{p}_i = n$)

$$\mathbf{p}(\hat{\lambda}) = (1.8082, 1.033, 1.2889, 1.3146, 0.4386, 0.5212, 0.6768, 0.8132,$$
$$0.9855, 0.4001, 1.6208, 0.9282, 1.1088, 1.0622)\,.$$

The resulting ranking of the members of the research group $\mathcal{N}$ is provided in Table 9. We infer that in view of the publications of the team members, the ranking according to EVP appears to be reasonable. In particular, the ranking according to EVP is similar to, yet different from, the ranking of the members according to their individual HB–scores disregarding the effects of cooperation. (The coefficient of correlation between EVP and the HB–score is 0.9675.)
